# Solvent Channels and Electric Fields Guide Proton Delivery to the Active Site of Heme Peroxidases

**DOI:** 10.1002/anie.202515743

**Published:** 2025-09-21

**Authors:** Reynier Suardíaz, Shakir Ali Siddiqui, Hanna Kwon, Marc W. van der Kamp, Lola González‐Sánchez, Peter C. E. Moody, Emma L. Raven, Adrian J. Mulholland

**Affiliations:** ^1^ Department of Physical Chemistry Complutense University of Madrid Madrid 28040 Spain; ^2^ School of Biochemistry University of Bristol University Walk Bristol BS8 1TD UK; ^3^ Center for Computational Chemistry School of Chemistry University of Bristol Cantock's Close Bristol BS8 1TS UK; ^4^ School of Chemistry University of Bristol Cantock's Close Bristol BS8 1TS UK; ^5^ Department of Physical Chemistry University of Salamanca Salamanca 37008 Spain; ^6^ Department of Molecular and Cell Biology and Leicester Institute of Structural and Chemical Biology University of Leicester Lancaster Road Leicester LE1 7RH UK; ^7^ Present address: Department of Molecular and Cell Biology and Leicester Institute of Structural and Chemical Biology University of Leicester Lancaster Road Leicester LE1 7RH UK

**Keywords:** DFT cluster model, Electric field, Heme peroxidase, Proton transfer, QM/MM, Solvent channel

## Abstract

The active sites of heme enzymes have evolved to control the formation of highly reactive intermediates in oxidative catalysis. Proton delivery to the heme is essential, yet the mechanisms of proton delivery remain poorly understood. Here, we identify routes and drivers of proton delivery in a heme peroxidase (ascorbate peroxidase) using computational approaches that combine classical, quantum, and hybrid methods with enhanced sampling and local electric field (LEF) analyses. Our results show that networks of active‐site water molecules facilitate proton exchange with Arg38, which may act as a transient proton carrier at the γ‐heme edge where the substrate binds. The distal His42 residue aids proton transfer into the active site via solvent at the δ‐edge. Molecular dynamics simulations of three heme peroxidases identify hydrated channels leading to both γ‐ and δ‐edges, allowing solvent protons to reach the active site. Comparison with eight other heme peroxidases shows that these channels are conserved. LEF analyses reveal a continuous electrostatic funnel drawing protons toward the heme from the γ‐ and δ‐edges, a feature that is broadly conserved across other peroxidases. These results suggest that nature pre‐organizes electrostatic funnels and solvent channels to provide multiple well‐defined routes for proton delivery in peroxidase catalysis.

## Graphical abstract

Electrostatic potential surfaces and electric field lines reveal that local fields guide proton delivery through multiple conserved solvent channels in heme peroxidases, enabling efficient access to the active site.

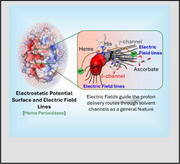

## Introduction

Heme‐containing enzymes are powerful and versatile catalysts. They are capable of a vast range of chemical oxidations, requiring only O_2_ or H_2_O_2_ and a source of electrons and protons. For H_2_O_2_‐dependent oxidations—namely in the heme peroxidases—the mechanisms of oxidation have been widely studied,^[^ ^1^, ^2^ ^]^ but the processes involved in proton delivery that accompany the O─O bond cleavage and the subsequent oxidation of substrate are still poorly defined. This matters because the movement of electrons in all heme enzymes is invariably linked to the movement of protons, and controlling both electrons and protons is key to the design of bespoke heme catalysts for specific applications in the future.^[^ ^3^ ^]^

The availability of structural data obtained using neutron diffraction for some heme peroxidase enzymes^[^ ^4^, ^5^ ^]^ has meant that the proton locations in the active site can now be directly visualized, because neutron crystallography, unlike X‐ray crystallography, can identify hydrogen (or deuterium) atom positions. These neutron structures reveal individual hydrogen bonds, including those involving active site water molecules. But while neutron diffraction can identify the positions of single hydrogen atoms (or, more specifically, deuterium atoms) in a static location, it cannot identify their origin, nor can it identify dynamic movements of protons during catalysis. While there have been computational studies on the reactivity of peroxidase and cytochrome P450 enzymes with H_2_O_2_, the focus has mainly been on formation of the critical Compound I intermediate and effects of water molecules.^[^ ^6^, ^7^, ^8^, ^9^, ^10^, ^11^, ^12^, ^13^, ^14^ ^]^ From this perspective, one could argue that the understanding of the dynamics involved in proton delivery in peroxidases has barely moved on since early ideas were presented 30 or so years ago.^[^ ^15^, ^16^, ^17^ ^]^

Here, we apply computational chemistry approaches to gain insights into the proton delivery mechanisms in a number of heme peroxidases. We evaluate potential mechanisms for proton supply from solvent into the active site of ascorbate peroxidase, and we identify solvent channels for proton delivery from the heme edge. We also analyze active site electric fields,^[^ ^18^, ^19^, ^20^ ^]^ and find that, in ascorbate peroxidase, the electric field is directionally shaped to favor and drive proton delivery along these channels. Analysis of other heme peroxidases shows that these solvent channels are conserved and that electrostatic preorganization along these channels is a general feature across the family.

## Results and Discussion

### Examination of Proton Movements in a Specific Proton Pathway

Structural information on proton delivery in a heme peroxidase comes from neutron crystallography work on ascorbate peroxidase (APX) in complex with its substrate ascorbate.^[^ ^21^ ^]^ In this case, a continuous pathway of hydrogen bonds from the substrate, bound at the γ‐heme edge, to the heme iron was identified. In this pathway, the conserved Arg38 residue plays a critical role but is observed in the neutron structure as a neutral (unprotonated) guanidine in contrast to the protonated guanidinium form that is typically considered the dominant state of arginine residues in proteins.^[^ ^22^ ^]^ However, several neutron structures have since been interpreted as showing neutral arginine side chains,^[^ ^23^, ^24^, ^25^ ^]^ as we also discussed in our previous work.^[^ ^21^ ^]^ Examination of hydrogen bonding in the crystal structure does not identify any feature that would favor deprotonation of Arg38. We therefore began by carrying out quantum mechanical calculations to assess the potential role of Arg38 in proton delivery.

**Figure 1:**
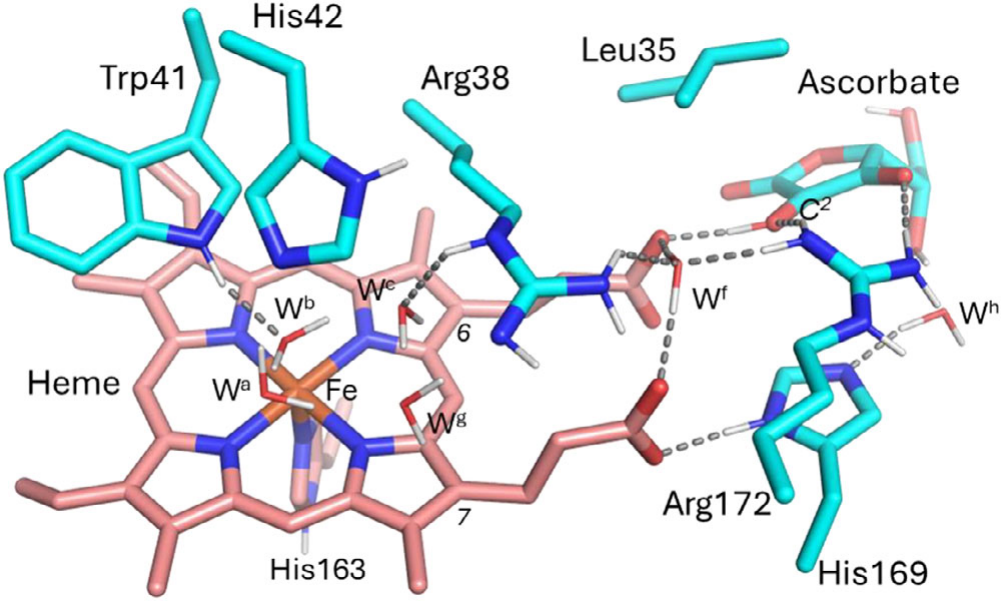
Schematic representation of the cluster model of APX used in DFT calculations here, showing key components of the main hydrogen bond network supporting proton transfers from the substrate to the heme. The cluster model contains the heme (pink), the proximal and distal histidine residues (His163 and His42), Arg38, Trp41, a water molecule coordinating to the Fe atom (W^b^), three other active site water molecules (W^a^, W^c^ beyond the previously considered cluster, and W^g^) and a water molecule (W^f^) that bridges the two propionate groups. The ascorbate substrate, which forms a hydrogen bond with the 6‐propionate group, was also included in the models along with Arg172, Leu35, His169, and a further water molecule (W^h^) bridging His169 and the ascorbate. The 6‐ and 7‐propionate groups are labeled. Hydrogen bonds are shown as dotted lines.

“Cluster” models^[^ ^26^, ^27^, ^28^, ^29^ ^]^ of key residues in the active site of APX, with Arg38 either neutral or positively charged, were investigated by density functional theory (DFT) calculations (Figure 1). The starting geometries for the cluster models used a tautomer of Arg38 as modeled in the neutron structure^[^ ^21^ ^]^ (see Section 1.1 in Supporting Information). We also considered other tautomers and all possible multiplicity states (see Figure S1 and Table S1). These residues and water molecules are the key components of the hydrogen bond network supporting proton transfer from the substrate to the heme during catalysis.^[^ ^21^ ^]^

We used DFT to calculate the proton affinity of Arg38 in the local environment of the protein, within the limitations of the cluster model. We first validated the level of theory using guanidine and arginine molecules, because experimental (gas phase) proton affinities for these molecules are available^[^ ^30^ ^]^ (see Section 1.2 in Supporting Information and Table S2 for details). Subsequently, we evaluated proton affinities using the same approach for Arg38 in the local protein environment of the active site using the cluster model, testing the effects of the environment and dielectric by using, respectively, a continuum solvent model of water and also an implicit solvent of *ε* = 4 (a typical value for the dielectric constant used to represent the effects of the protein environment in enzyme studies using DFT cluster models^[^ ^26^, ^31^, ^32^ ^]^). The proton affinities in continuum solvent or protein model are lower in absolute value for all the species, but the trends and conclusions are similar (see Section 1.2 in Supporting Information). These calculations indicate that the local active site structure surrounding Arg38 does not significantly lower the proton affinity (Table 1) and that the protonated form of Arg38 is substantially more stable than the neutral form, suggesting that the neutral form of Arg38 as identified in the neutron structure may reflect a transient state rather than the most populated form under physiological conditions.

**Table 1:** Proton affinities of guanidinium, arginine (alone), and Arg38 (kcal mol^−1^).^a)^

	Guanidine	Arginine	Arg38 model
Relative proton affinity	74.2	81.9	137.0

^a)^ As defined by system‐neutral + H_3_O^+^→system‐charged + H_2_O (see Eq. 3 in the Supporting Information) at the B3LYP‐D3/6‐31G(d,p) level of theory in the gas phase.

We then applied QM/MM calculations^[^ ^33^ ^]^ at the DFT QM level to further test the effects of the extended protein environment on proton affinity and to quantify specific proton movements to Arg38. The DFT/MM‐optimized geometries (derived from MM molecular dynamics (MD) simulation snapshots) for both the neutral and protonated Arg38 systems closely resemble the crystal structures, with an RMSD of approximately 0.6 Å for heavy atoms (they are also similar to the geometries taken from MM MD, indicating that the MM geometries are reasonable). DFT/MM Mulliken charges and spin densities, with and without the MM point charges reveal no significant differences, suggesting that the extended protein environment beyond the previously considered cluster does not significantly polarize the reactive center. The main differences between DFT/MM geometries and the crystal structure are in the positions of active site water molecules, which move and reorient and are dependent on the starting frame of the MD.

**Figure 2:**
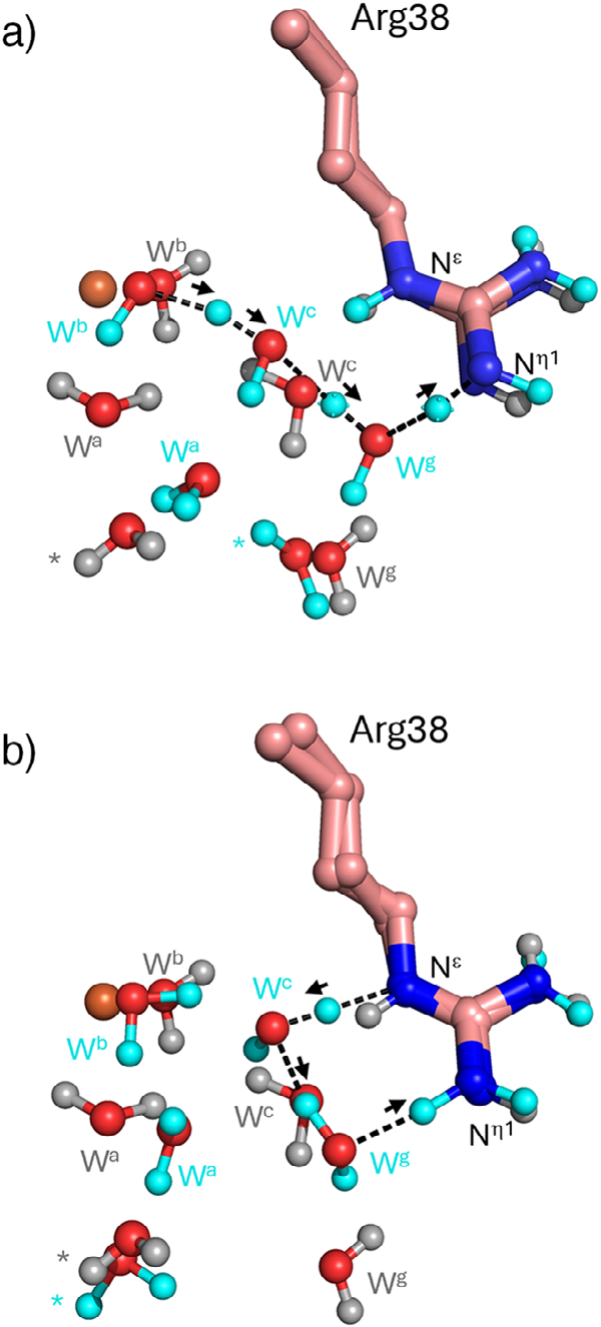
Overlay of experimental (neutron structure, PDB 6XV4)^[21]^ and computational (DFT/MM) models, showing Arg38 and active water molecules. Polar hydrogen atoms are depicted as smaller spheres (gray for crystal positions and cyan for final positions after DFT/MM geometry optimization); iron is shown as an orange sphere. a) DFT/MM geometry optimization, starting from the neutron structure model, results in protonation of Arg38 via proton transfer from the iron‐bound water molecule (W^b^) through a chain of three water molecules (W^b^, W^c^, W^g^). A proton, initially on W^g^, forms a bond with the N^η1^ atom, generating a positively charged arginine. Subsequently, W^g^ abstracts a proton from W^c^, which then pulls a proton from W^b^. b) DFT/MM geometry optimization, starting from the neutron structure (with Arg38 as tautomer 1; see Section 1.1 in Supporting Information), leads to formation of a different tautomer (tautomer 3; see Section 1.1 in Supporting Information) through proton exchange with W^c^ and W^g^. A proton transfers from the N^ε^ of Arg38 to W^c^, and another proton shifts from W^g^ to the N^η1^ of Arg38. In both (a) and (b), black arrows indicate movements of atoms during geometry optimization. Water molecules involved in these processes occupy positions consistent with the crystallographic waters. Notably, W^g^ in (a) shifts significantly toward Arg38. Videos (see Movies S1 and S2) provide detailed visualization of movements during geometry optimizations.

Using the same procedure as for the DFT cluster structures, but applied to several DFT/MM structures, we calculated proton affinity values, all of which clearly favor the protonated form of Arg38 (see Section 1.3 in Supporting Information and Table S4). Notably, DFT/MM geometry optimizations of some selected snapshots led to significant structural rearrangements when starting from a neutral Arg38, and in several cases, spontaneous protonation of Arg38 through a succession of coupled proton transfers. These results provide direct insight into the specific proton movements along this proton transfer pathway. In one instance, Arg38 received a proton from a neighboring water molecule (W^g^, Figure 1, treated as QM). W^g^ then pulled a proton from a second water molecule (W^c^), which in turn received a proton from the iron‐bound water molecule (W^b^) (Figure 2a). These calculations provide evidence for proton transfer from the distal water molecule (W^b^) to Arg38, leading to protonation of Arg38 at the expense of W^b^ (which forms a ferric‐hydroxide species). DFT/MM optimization of a different frame led to spontaneous transformation of the neutral Arg38 from tautomer 1 to tautomer 3 (see Figure S1), facilitated by nearby water molecules. These DFT/MM calculations show that when W^c^ and W^g^ occupy the positions depicted in Figure 2b, transfer of a proton from the N^ε^ of Arg38 through the water chain to the N^η^ of Arg38 is favored, converting Arg38 from one tautomer to another. DFT/MM optimized structures and a video showing each process are provided in the Supporting Information.

Although the proton affinity calculations suggest that the neutral state of Arg38 is only transient, the QM/MM calculations provide direct evidence of a proton transfer network connecting Arg38 and the distal water (Figure 2a). Arg38 is able to accept and donate protons and can transition between different tautomeric forms (Figure 2b). This highlights the importance of active site water molecules. Also, the water molecules must be correctly arranged for proton transfer to take place: only if they are appropriately positioned and oriented can proton transfers occur.

### Identification of Proton Delivery Channels

We next performed MD simulations of APX with Arg38 in protonated and neutral states to examine the solvent accessibility of the active site and to identify other possible proton channels. For comparison, we also carried out equivalent MD simulations on two other ferric peroxidases: cytochrome *c* peroxidase (C*c*P) and horseradish peroxidase (HRP) with protonated Arg38 or equivalent. In addition, we used CAVER 3.03,^[^ ^34^, ^35^ ^]^ to analyze both the crystal structures and the MD trajectories of these systems. CAVER was used to detect possible internal channels based on geometrical criteria, and the MD simulations assessed whether these channels are sufficiently hydrated and structurally stable to permit water, and thus proton, transit under dynamic conditions.

The MD simulations of APX, irrespective of the protonation state of Arg38, reveal a well‐hydrated heme active site, with an average of approximately five water molecules within two 8 Å spheres centered on the N^η1^ of Arg38 and the Fe atom. Water molecules occupy positions consistent with the neutron structures; they exchange with each other on the timescale of the simulations. Our analysis identified two distinct channels, δ‐ and γ‐channels, that could potentially facilitate proton transfer to the active site, as described below.

**Figure 3:**
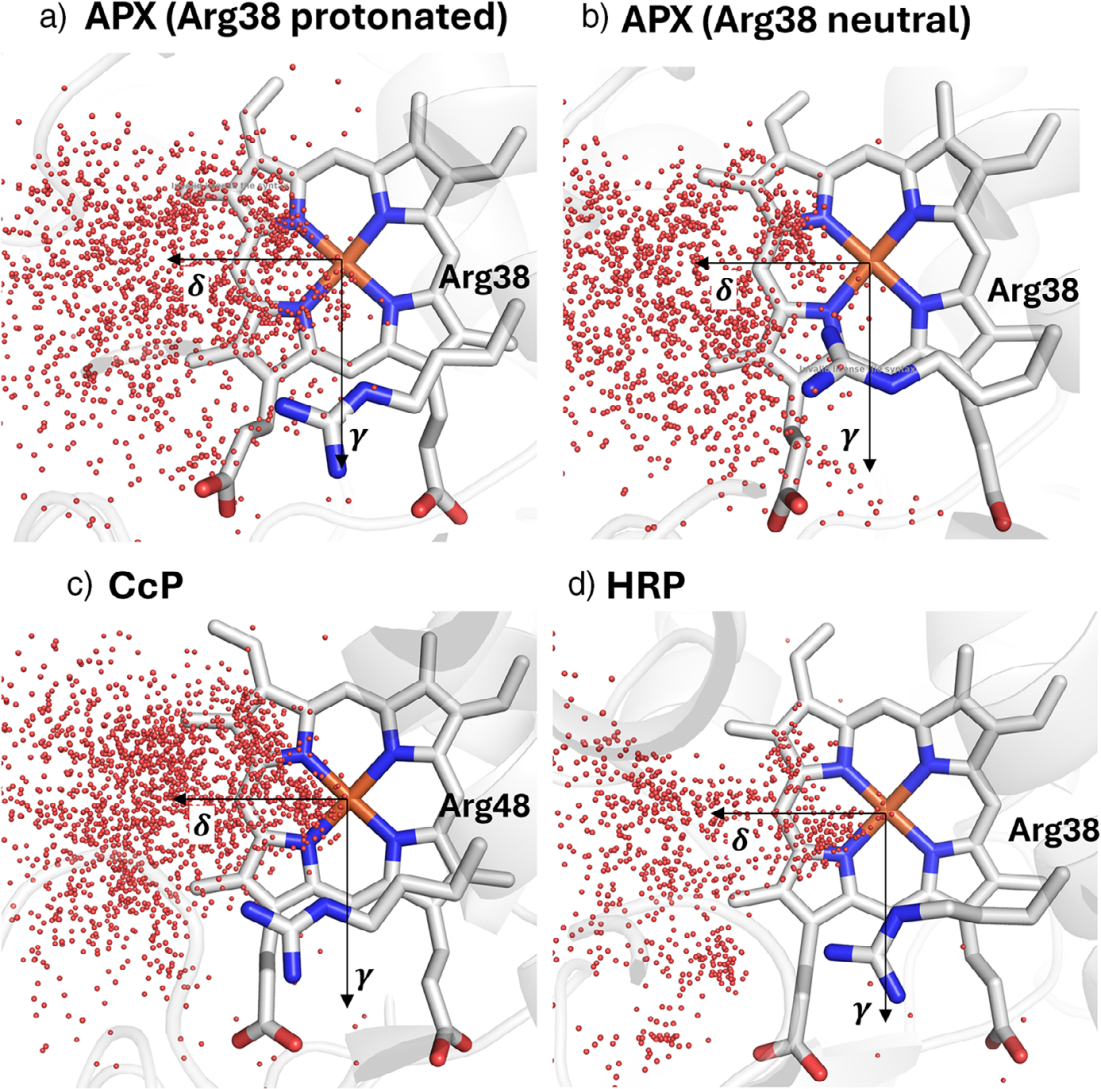
Positions of water molecules from MD snapshots, showing structures containing water wires (≥5 consecutive hydrogen‐bonded water molecules) extending from the heme reaction center. The δ‐ and γ‐directions are indicated in each panel, demonstrating the preferential formation of water chains/wires along the δ‐direction. Shown are a) APX with protonated Arg38 (PDB 6TAE^[^ ^21^ ^]^), b) APX with neutral Arg38 (PDB 6XV4), c) CcP (PDB 4CVI^[^ ^4^ ^]^), and d) HRP (PDB 1H5A^[^ ^36^ ^]^). Hydrogens are omitted for clarity.

**Figure 4:**
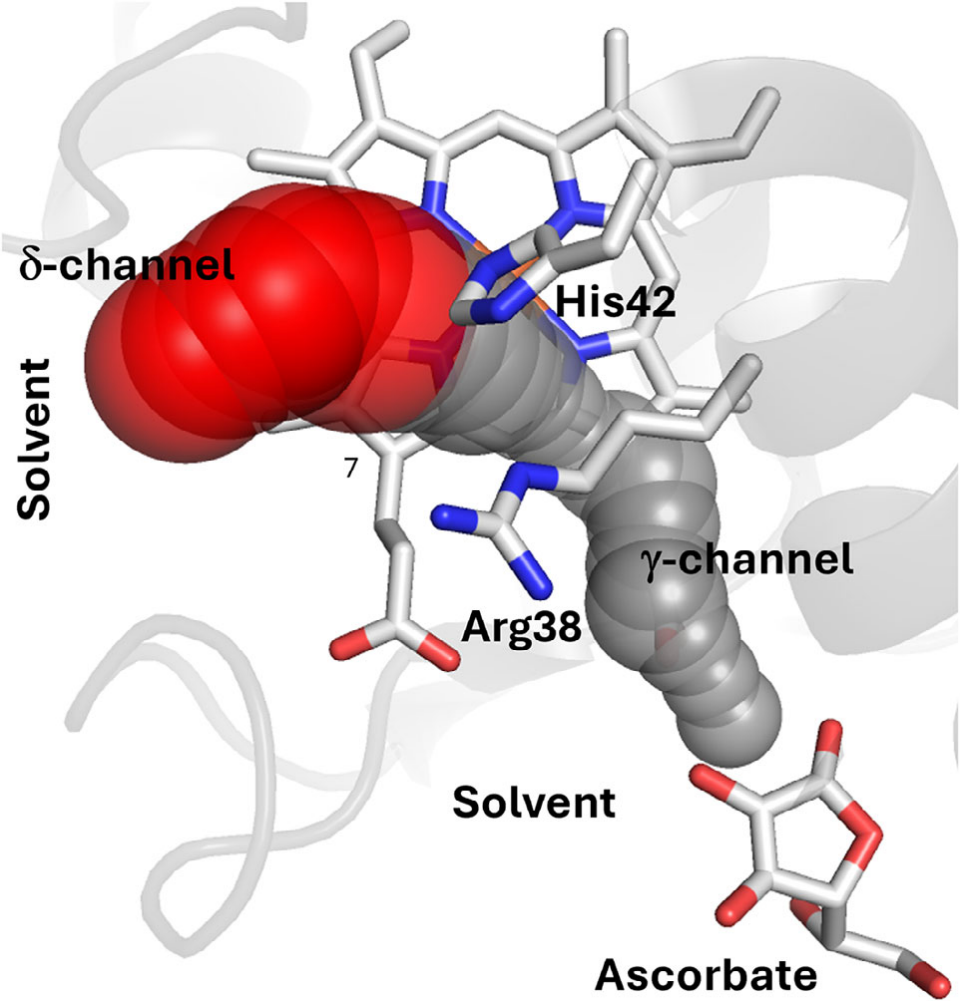
Solvent‐accessible channels identified in the 6TAE^[21]^ crystal structure of ascorbate peroxidase, originating from the center of the active site and extending to the solvent at the δ‐ (in red) and γ‐ (in gray) heme edges. The channels are identified by CAVER.^[^ ^37^ ^]^

δ‐Channel: The MD simulations of APX reveal multiple clear, unbroken chains of consecutive (hydrogen‐bonded) water molecules forming a channel from the surface solvent at the δ‐heme edge to the center of the active site (defined as the oxygen atom coordinating the heme iron) (see Figure 3a,b; Sections 1.5 and 1.6 in Supporting Information). These consecutive water chains align well with the red channel shown in Figure 4, identified by CAVER in the δ‐direction as above, see also Figure S3. This channel is detected using both the default 0.9 Å probe in CAVER and with an increased probe radius of 1.4 Å, which approximates the van der Waals radius of a water molecule. This suggests that there is sufficient space along the channel to allow for the presence and dynamic exchange of water molecules, rather than a strictly single‐file arrangement. This is consistent with the water networks observed in the MD simulations, which could support proton transfer via an extended hydrogen‐bonded network. This channel, which allows exchange of water molecules between the heme iron and the solvent, is conserved across other peroxidases (C*c*P and HRP, Figure 3c,d), as our simulations and analyses show.

Taking the channel identification and MD simulation results together indicates that protons can flow between protein surface and the heme iron, but the data does not provide information about the directionality of this flow nor the driving force.

We then tested the potential proton pathway and the direction of transfer through the solvent channel in the δ‐direction, identified in red in Figure 4 using QM(DFTB2)/MM‐Umbrella Sampling reaction simulations (see Section 1.7 in Supporting Information for details). These simulations showed the transfer of a proton from the solvent (at the δ‐heme edge) to His42, as shown in Figure S5. This transfer is independent of the presence of bound ascorbate or the protonation state of Arg38, as these are not included in the QM part of the calculations and are located far from the proton pathway. Notwithstanding the limitations of classical dynamics (such as lack of quantum tunneling and zero‐point energy) for treatment of proton transfers, these simulations show that the transfer of a proton from the distant solvent at the δ‐heme edge to His42 is favored (see Section 1.7 in Supporting Information and Figure S5).

γ‐Channel: Looking at proton delivery in the direction of the γ‐heme edge, a continuous chain of water molecules from the protein surface at the γ‐heme edge to the heme is rarely observed in our MD calculations, although it is observed in a few isolated frames (see Figure 3a,b). However, water chains are frequently observed on both sides of Arg38 (one side leading to the heme iron and the other toward the γ‐edge of the protein). These form potential proton pathways composed of water molecules and Arg38 (regardless of the protonation state of Arg38). CAVER only identifies a 1.4 Å probe channel in rare frames where temporary protein movements allow a water chain to form, but this is uncommon. However, when using a 0.9 Å probe, a solvent channel is consistently detected in the γ‐direction (see gray channel in Figure 4). This suggests that although the passage of water molecules is restricted in this direction, proton transfer via a Grotthuss mechanism is possible through a single‐file chain of water molecules and polar residues such as Arg38.^[^ ^38^ ^]^

### Directionality of Proton Delivery in APX

We further assessed the directionality of proton movements in these channels using calculations of local electric field (LEF). LEF quantifies the local electric field in a protein based on the charge distribution and is known^[^ ^39^ ^]^ to be a useful tool for quantifying proton delivery mechanisms. This is because electric field vectors are directed from regions of positive charge to negative charge and positively charged protons move in the same direction as the electric field (positive to negative). This means that quantification of the local electric field can report on the equivalent movement of protons and can provide information on the direction.^[^ ^39^, ^40^ ^]^

**Figure 5:**
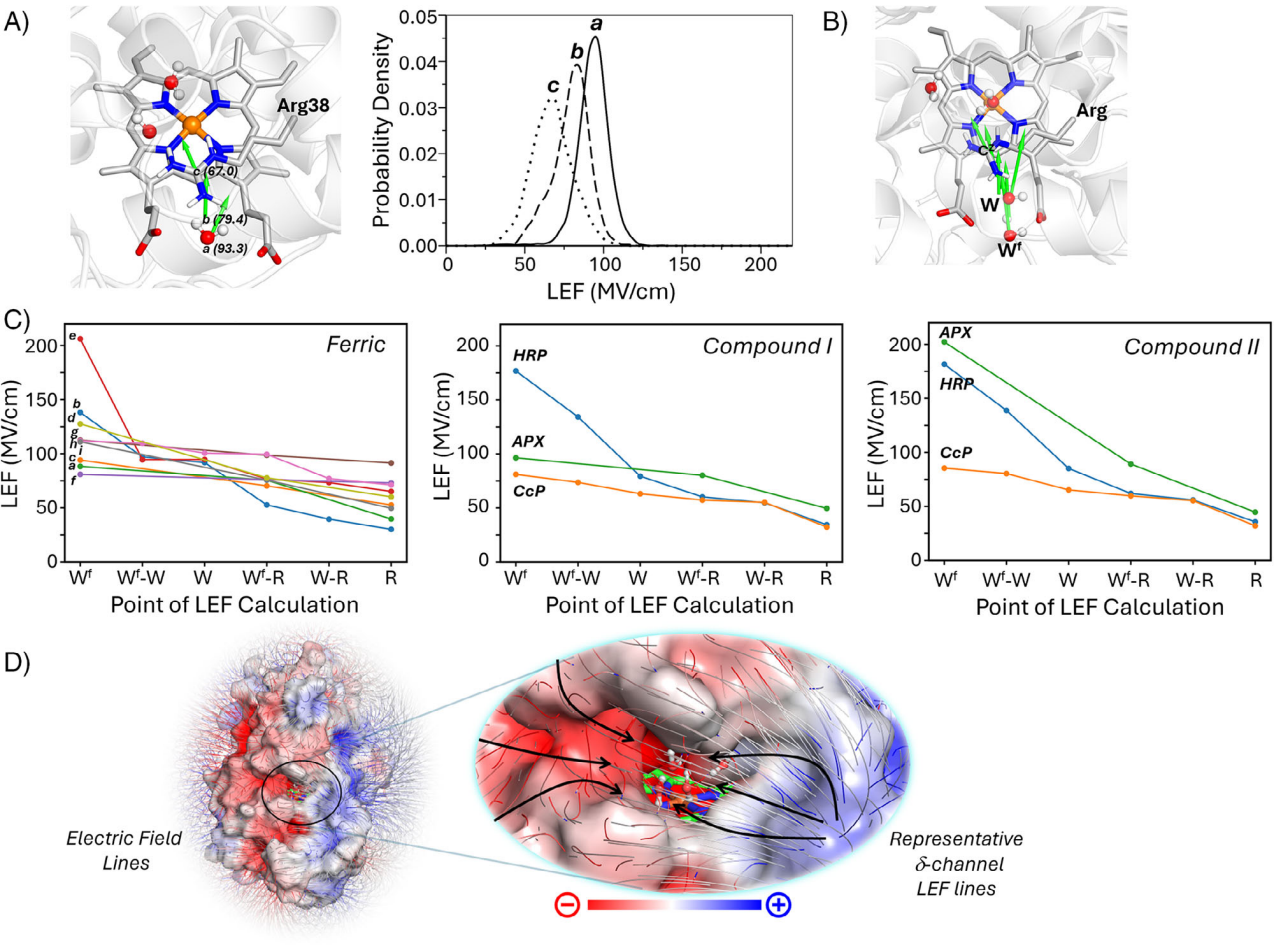
Local electric fields (LEFs) in APX and other heme peroxidases. A) Left: LEF along the γ‐channel in APX at three key points: a) at the location of the water molecule W^f^, b) at the midpoint between W^f^ and the C^z^ atom of Arg38, and c) at the C^z^ atom of Arg38. The orientations of the electric field vectors at each key point are shown in green arrows. Right: The plot of probability density shows the distribution of LEF magnitudes during the MD simulation; note the comparison with the same plots for neutral Arg38 in Figure S6. B) Representative structure of the γ‐channel for all other heme peroxidases considered, showing the conserved active Arg residue (analogous to Arg38 in APX) and the equivalent water molecules along the proton transfer pathway. The green arrows show the points at which the electric fields were computed and indicate the direction and relative magnitude of LEF vectors toward the heme iron. C) Left: LEF values calculated at different points along the γ‐channel (shown in panel B), as above for the ferric state of a) APX (PDB code 6XV4^[^ ^21^ ^]^), b) HRP (1ATJ^[^ ^41^ ^]^), c) C*c*P (3R98^[^ ^42^ ^]^), d) MnP (3M5Q^[^ ^43^ ^]^), e) DyP (6KMN^[^ ^44^ ^]^), f) LiP (1B85^[^ ^45^ ^]^), g) MPO (1DNU^[^ ^46^ ^]^), h) LPO (5B72^[^ ^47^ ^]^), i) ARP (1ARP^[^ ^48^ ^]^). Middle: the equivalent plots for the Compound I intermediates of APX (2XI6^[^ ^5^ ^]^), HRP (1HCH^[^ ^36^ ^]^), and C*c*P (5EJT^[^ ^49^ ^]^). Right: the equivalent plots for crystal structures of the Compound II intermediates of APX (PDB code 5JPR^[^ ^5^ ^]^), HRP (1H55^[^ ^36^ ^]^) and CcP (2XJ5^[^ ^50^ ^]^). The quantitative data for LEF values corresponding to all these structures is provided in Table S7. D) Electric field lines across the APX enzyme surface (left) and a zoomed‐in view of a representative δ‐channel (right) highlighting the electric field lines travelling from the positively to the negatively charged region. Red and blue contours indicate regions of negative and positive electrostatic potential, respectively, while arrows depict a schematic representation of electric field lines near the δ‐channel, illustrating LEF directionality toward the active site. The right‐hand side show the zoom of the active site; the field lines point from positive to negative, some of these positive regions are outside the zoomed area.

We first investigated whether the γ‐channel is electrostatically preorganized and whether this affects the directionality of potential proton transfer. We quantified the LEF along the γ‐channel in APX for the protonated state of Arg38 and the three neutral tautomers shown in Figure S1. We used LEFs to measure the electrostatic gradient and directionality at three key points along the γ‐channel between the protein surface and the heme iron (points a–c in Figure 5A): a) at the location of the water molecule W^f^; b) at the midpoint between W^f^ and the C^z^ atom of Arg38; and c) at the C^z^ atom of Arg38. A clear decrease in the electric field magnitude was observed in a direction moving from the surface toward heme iron (points a to c), (Figure 5A). A similar decrease in the electric field magnitude in the direction of the heme was also observed for the neutral Arg38 tautomers, though with slightly different field magnitudes due to the altered charge of neutral Arg38 (Figure S6). The effect of this electric field gradient, in terms of proton movement, is that there is an electrostatic funnel running from positive to negative in the direction from the surface toward the heme iron, which guides protons along the γ‐channel from the protein surface to the heme iron. This steep gradient and vector orientation were consistent across all MD snapshots. These results not only reinforce the γ‐channel's role in directing proton migration but also show the driving force for transfer is directed along this channel. This focusing and direction of the electric field appears to be a mechanism to support proton transfer along the γ‐channel. The consistent directionality from the protein surface to the heme iron suggests that the electric fields within the protein are evolutionarily tailored to promote charge migration to the active site in APX. The potential generality of this concept across other peroxidases is explored below.

### Proton Channels in Other Peroxidases

To explore whether the electrostatic preorganization observed in the γ‐channel of APX is a common feature among other heme peroxidases, we applied LEF analysis to several other enzymes in the family (HRP, C*c*P, manganese peroxidase (MnP), dye decolorizing peroxidase (DyP), lignin peroxidase (LiP), myeloperoxidase (MPO), lactoperoxidase (LPO), and *Arthromyces ramosus* peroxidase (ARP)). This analysis also included structures where the position of the conserved distal arginine varies across these enzymes and captured both “in” and “out” conformations of the distal arginine (see Supporting Information and Table S6). Using crystal structures of each peroxidase, we identified the γ‐channel based on structural alignment of their ferric structures with ferric APX and then calculated the LEF along the channel in the same way as for APX (Figure S7). Across all peroxidases considered, we observed clear decrease in the magnitude of the electric field along the γ−channel, with LEF magnitudes decreasing in the direction of the heme active site (Figure 5C and Table S7). Moreover, these LEF vectors (Figure 5B) are uniformly oriented from the surface toward the heme iron, forming a coherent electrostatic funnel analogous to that observed in APX. This was also observed when examining the crystal structures of both the Compounds I and II intermediates of APX, HRP, and CcP (Figure 5C). Notably, while the direction of the electric field was consistent across all the heme peroxidases and oxidation states examined, the magnitudes and gradients of the fields varied, suggesting that tuning of the electric field may influence the efficiency of proton delivery and reflect functional adaptations within different peroxidases.^[^ ^51^, ^52^ ^]^

The LEF analysis above reveals a consistent electrostatic funnel guiding proton movement, highlighting an electrostatic preorganization across the family of peroxidase enzymes. To understand the spatial arrangement of these fields within the context of the protein structure, visualization of the electric field lines is very useful: this can reveal how the electric field lines are organized to direct proton movements. The presence of both γ‐ and δ‐channels for proton delivery, as identified for APX in Figure 4, was also identified in all of the other peroxidases that we examined (Figure S7). Notably, electric field lines near the δ‐channel in all cases are arranged in a way that guides water molecules into the cavity, which is consistent with the results from QM(DFTB2)/MM‐umbrella sampling and MD simulations (see Figure S5 and Section 1.7 in Supporting Information). For the δ‐channel, this is easily visualized in a 2D format (Figure 5D), as the δ‐heme edge in peroxidases is exposed to solvent and the electric field lines leading into the heme active site are clearly observed for all peroxidases examined (Figure S8A). Such organization of the electric field lines is less easily visualized in the equivalent 2D view of the γ‐channel, because the 2D view cannot convey the spatial arrangement of electric field lines through the interior of the protein (Figure S8B). We therefore used an immersive 3D visualization platform (UnityMol)^[^ ^53^ ^]^ to render the electric field lines within a virtual–reality‐compatible environment. This immersive approach allows the viewer to travel inside the protein to trace the paths of electric field lines from the γ‐channel entrance down to the active site. The Movies S3–S5 show the paths of these electric field lines for both the γ‐ and δ‐channels for three representative peroxidases (APX, HRP, and C*c*P). These 3D immersive visualizations confirm that the electric field lines extend from the surface toward the iron in both channels, forming a continuous electrostatic funnel to pull protons through the channels toward the iron.

## Discussion

All oxidative heme enzymes use oxygen (O_2_) or its reduced equivalent hydrogen peroxide (H_2_O_2_) to form reactive ferryl intermediates that are the basis of their oxidative power.^[^ ^1^, ^2^ ^]^ These intermediates, common to all known heme oxidative enzymes, have been studied since the 1930s.^[^ ^54^, ^55^ ^]^ Significant progress has been made in identifying their structures and reactivity.^[^ ^14^, ^56^ ^]^ Many decades have passed since the discovery of the ferryl intermediates, but the proton transfer mechanisms associated with O─O bond cleavage and the subsequent re‐reduction of heme by reducing substrate(s) are still largely unknown in many cases. In particular, the precise origin of proton(s)—whether they originate from active site residues, the substrate, or elsewhere—has not been generally established. Similarly, the role of water molecules, within the active site and linking to solvent, as potential conduits for proton migration as well as their points of entry and exit during the catalytic cycle, is not well understood. Even in cases where continuous hydrogen bonding pathways have been identified as a plausible route for proton delivery (such as heme oxygenase, cytochrome c oxidase, cytochrome P450, MauG, cytochrome *bc*1 and APX^[^ ^1^, ^2^, ^21^, ^57^, ^58^, ^59^, ^60^, ^61^, ^62^ ^]^), it has been unclear whether these are broadly representative of other heme enzymes.

One of the most significant findings in this work is that QM(DFT)/MM calculations show the feasibility of dynamic proton interchange between a conserved active site arginine in APX, Arg38, and surrounding water molecules, also facilitating the interconversion of Arg tautomers in the neutral form (Figure 2b). The calculations also show that a neutral Arg38 can accept protons depending on the local instantaneous conditions (Figure 2a). The results show that the guanidine group can alter its tautomeric state to support dynamic proton transfer events in an active site in which locations of water molecules remain approximately constant. This is consistent with a role for Arg38 in proton delivery from the γ‐heme edge to the heme. To act in this way, Arg38 would need to interconvert between protonated (guanidinium) and neutral (guanidine) forms as part of a proton transfer network connecting the substrate to the heme. Neither the DFT cluster models nor the QM/MM calculations show lowering of the *p*K_a_ of Arg38, so a neutral form of Arg38 is likely to exist only transiently as part of a solvent‐mediated proton transfer pipeline. There is increasing evidence that arginine can function as an acid and exist at least transiently in proteins under physiological conditions.^[^ ^25^, ^63^, ^64^, ^65^ ^]^ We note that in another tetrapyrrole‐binding protein (the photosensor protein RcaE)^[^ ^66^ ^]^ an active site lysine residue has been shown to be neutral, and to switch between protonation states. The substantive point in the case of APX (and potentially other peroxidases) is that results from several different methods show that movement of protons via active site networks, including water and via Arg38, is mechanistically feasible.

In terms of proton channels, the MD and QM(DFTB2)/MM calculations, clearly show the existence of a stable, continuous, proton transfer channel connecting the solvent, at the δ‐heme edge, to the heme (Figure 4) in all peroxidases examined. This pathway includes a well hydrated channel, containing water molecules extending from the center of the active site to solvent, with His42 playing an important role as a switching residue for controlling the proton supply (Figure S5). There is strong evidence that substrates bind at multiple locations in heme peroxidases,^[^ ^67^, ^68^, ^69^ ^]^ including at the δ‐heme edge and potentially other surface locations. Effective proton delivery mechanisms are required to complete the oxidation reaction. Our observations provide evidence for proton delivery mediated by water molecules from the δ‐heme edge that is relevant across the heme peroxidase family (all of which oxidise organic substrates). The CAVER analysis and MD simulations also identify a second proton channel to the γ‐heme edge (Figure 4). This provides two possibilities for delivery of protons to the active site in heme peroxidases and we interpret this to mean that both solvent channels are generically useful.

LEF analysis shows that both the γ‐ and δ‐channels can contribute to proton movement, in APX and in other peroxidases. The shaping of the electric field to drive proton transfer along the solvent channels appears to be a general feature of peroxidases and is likely to be important in other similar proteins. The coherent direction and close packing of electric field lines in the δ‐channel show a well‐arranged electrostatic environment that facilitates water‐mediated proton transfer (see Figure S8A). The γ‐channel displays a strong, directional electric field pointing toward the heme iron from protein surface (see Figure 5 and Section 1.9 in Supporting Information). Together, these observations suggest that the electric fields are electrostatically preorganized along both solvent channels to support proton delivery. These results underscore the broader role of electrostatics in guiding charge—here, proton migration. They also emphasize how VR‐enhanced 3D visualization offers intuitive spatial insights that are often obscured in traditional 2D representations^[^ ^70^ ^]^ (see illustrative Movies S3–S5).

Our findings are consistent with other evidence across the heme enzyme family (e.g., for cytochrome P450^[^ ^40^, ^71^ ^]^) showing that local electric fields are key determinants of catalytic efficiency by modulating and defining proton and electron transfer pathways and stabilizing reaction intermediates. Our findings also chime with observations on other enzymes that show that electric fields in proteins are optimized by evolution for specific catalytic functions.^[^ ^72^, ^73^ ^]^ Similar hydration‐controlled proton transfer mechanisms have been reported in other redox enzymes, such as cytochrome c oxidase and respiratory Complex I, where changes in hydration and electric fields gate proton delivery during catalytic cycles. In these systems, hydration of internal cavities based on conformational dynamics and electrostatic preorganization has been shown to guide proton movement in a manner conceptually analogous to our findings.^[^ ^39^, ^74^, ^75^, ^76^, ^77^ ^]^

## Conclusions

The source of the two protons needed for reduction of the heme during peroxidase catalysis has typically been assumed to originate from the substrate because peroxidase substrates are considered, formally, as hydrogen atom donors (i.e., providing an electron and a proton). Our results show that this model of proton‐coupled electron delivery is an over‐simplification that does not represent the true picture in the context of a dynamic, solvated heme enzyme in which substrates are located on the surface rather than within hydrogen bonding distance of the heme. Instead, we demonstrate here the existence of open solvent channels, through which the required protons can be delivered from solvent. Further, we find that the electric field in the protein is shaped and focused to guide protons along these solvent channels into the active site, and that this is a feature of all the peroxidases examined. Overall, this model for proton delivery appears to be quite general and is potentially relevant for design and engineering of natural and de novo heme enzymes.

## Supporting Information

Details of DFT, QM(DFT)/MM calculations, MM MD simulations, QM(DFTB2)/MM MD simulations, and LEF calculations and visualization; Tables S1–S7; Figures S1–S8. Excel file containing a dataset of Mulliken charges and spin densities for each atom in the DFT/MM‐optimized structures, calculated with and without the MM point charges and the respective differences: polarization.xlsx. This dataset reveals that polarization effects from the distant protein environment have minimal influence on the reaction center's electronic and structural properties. Complementary videos for Figure 2 (Movies S1 and S2) and for the local electric field lines visualization in VR (Movies S3–S5). Coordinates of the DFT‐optimized largest cluster models and QM/MM‐optimized structures for different tautomers and spin multiplicities. Custom analysis scripts for identifying consecutive water chains in MD.

## Conflict of Interests

The authors declare no conflict of interest.

## Supporting information



Supporting Information

Supporting Information

Supporting Information

Supporting Information

Supporting Information

Supporting Information

Supporting Information

Supporting Information

Supporting Information

## Data Availability

The data that support the findings of this study are available in the Supporting Information of this article.
