# Supplementary material for: Solvent Channels and Electric Fields Guide Proton Delivery to the Active Site of Heme Peroxidases
Source: Angew Chem Int Ed Engl. 2025 Sep 21;64(50):e202515743. doi: 10.1002/anie.202515743 (PMC12684355; doi:10.1002/anie.202515743)
Supplement: Supplementary file 1 — Supporting Information [file ANIE-64-e202515743-s005.docx]

Supporting Information for:

Solvent Channels and Electric Fields Guide Proton Delivery to the Active Site of Heme Peroxidases

Reynier Suardíaz,^[a,b,c,#]*^ Shakir Ali Siddiqui,^[c,#]^ Hanna Kwon,^[d,†]^ Marc van der Kamp,^[b]^ Lola González-Sánchez,^[e]^ Peter C. E. Moody,^[f]^ Emma L. Raven^[d]*^ and Adrian J. Mulholland^[c]*^

[a] Department of Physical Chemistry, Complutense University of Madrid, Madrid, 28040, Spain
E-mail: reysuard@ucm.es

[b] School of Biochemistry, University of Bristol, University Walk, Bristol, BS8 1TD, UK

[c] Centre for Computational Chemistry, School of Chemistry, Cantock’s Close, University of Bristol, Bristol, BS8 1TS, UK

[d] School of Chemistry, Cantock’s Close, University of Bristol, Bristol, BS8 1TS, UK

[e] Department of Physical Chemistry, University of Salamanca, Salamanca, 37008, Spain

[f] Department of Molecular and Cell Biology and Leicester Institute of Structural and Chemical Biology, University of Leicester, Lancaster Road, Leicester, LE1 7RH, UK

E-mail: [reysuard@ucm.es](mailto:reysuard@ucm.es), [emma.raven@bristol.ac.uk](mailto:emma.raven@bristol.ac.uk), adrian.mulholland@bristol.ac.uk

^#^These authors contributed equally to this work.

^†^ Present address - Department of Molecular and Cell Biology and Leicester Institute of Structural and Chemical Biology, University of Leicester, Lancaster Road, Leicester, LE1 7RH, UK

Table of Contents

[1 METHODS AND ANALYSIS 2](#_Toc199342626)

[1.1 DFT calculations. 2](#_Toc199342627)

[1.2 Proton affinities. 3](#_Toc199342628)

[1.3 QM(DFT)/MM calculations. 4](#_Toc199342629)

[1.4 Molecular dynamics (MD) simulations. 5](#_Toc199342630)

[1.5 Identification of proton delivery channels. MD Analysis. 6](#_Toc199342631)

[1.6 Identification of proton delivery channels. Channel (CAVER) Analysis. 7](#_Toc199342632)

[1.7 QM(DFTB2)/MM calculations. 8](#_Toc199342633)

[1.8 Local electric field (LEF) calculations. 9](#_Toc199342634)

[1.9 Visualisation of electric field lines in virtual reality (VR). 10](#_Toc199342635)

[1.10 Coordinates 13](#_Toc199342636)

[1.11 References 13](#_Toc199342637)

# 1 METHODS AND ANALYSIS

1.1 DFT calculations. Starting geometries for cluster models were taken from the neutron crystal structure of ascorbate peroxidase in complex with ascorbate^[1]^ (PDB 6XV4), which corresponds to tautomer 1 in Figure S1. The neutral and protonated starting geometries of the cluster models only differ by their net charge and the presence of a single proton on Arg38. The RMSD between neutron structures which have a neutral Arg38 (ferric APX-ascorbate complex, PDB 6XV4 (data collected at 100K))^[1]^ and a protonated Arg38 (ferric APX, PDB 6TAE (data collected at 100K))^[1]^, calculated considering the components used in the cluster models, is 0.2 Å. Use of the same starting structure for the neutral and protonated calculations (differing only by the single proton on Arg38) is thus considered valid. Amino acids were truncated at Cα, so that only Hα and side chains were kept in the model. All Cα atoms were kept frozen in their positions as defined in the neutron structure of the ferric APX-ascorbate complex during DFT calculations to preserve the spatial arrangement of the active site residues. Calculations were performed using the B3LYP^[2]^ functional as implemented in the Gaussian 09 package.^[3]^ Dispersion corrections were included using the B3LYP-GD3BJ method (D3 version of Grimme’s dispersion with Becke-Johnson damping).^[4]^ The 6-31G(d,p) basis set was employed for all the atoms except for Fe, for which the LANL2DZ basis/effective core potential was used, as in previous work.^[5-9]^ As the protein is in its resting ferric state, the possible spin states of the heme group have multiplicities of M = 2, 4 and 6, all of which were considered. Different tautomers for the neutral form of Arg38 were also considered, as depicted in Figure S1. Tautomers 1 and 3 have been noted previously.^[10]^ Tautomer 2 invokes breaking of the hydrogen bond between N^η2^ of Arg38 with the bridging water; this would need a reorganization and further relaxation of the geometry that was not included in the calculation, which explains the higher relative energy values of tautomer 2, as shown in Table S1.

The DFT calculations corrected an anomalous orientation of the hydrogen atom in the hydrogen bond between the C^2^-OH of ascorbate and the 6-propionate. In the DFT-optimized geometry, the hydrogen atom of the C^2^-OH group of the ascorbate forms a nearly 180^o^ angle with the oxygen atom of the 6-propionate, see Figure S2A, consistent with a strong hydrogen bond.

DFT geometry optimizations showed the system to be more stable with multiplicities M=2 and M=6; M=4 is notably less stable (Table S1). All the DFT optimized geometries are very similar, and very similar to crystal structures. For instance, at M=2, the RMSD is 0.3 Å between neutral and charged arginine systems, excluding hydrogen atoms and water, as shown in Figure S1. The most important difference between the neutral and charged Arg38 systems is that water molecules slightly reorientate in the active site (compare the neutral, Figure S2A, and protonated, Figure S2B, states of Arg38).

| 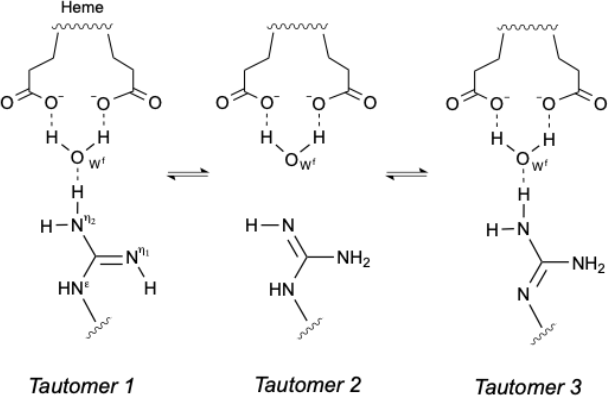 |
| --- |
| **Figure S1.** Tautomers of neutral Arg38 and its relative position in relation to the heme propionate groups and the conserved bridging water molecule (W^f^) observed in the crystal structure. Tautomer 1 is observed in the crystal structure.^[1]^ |

| 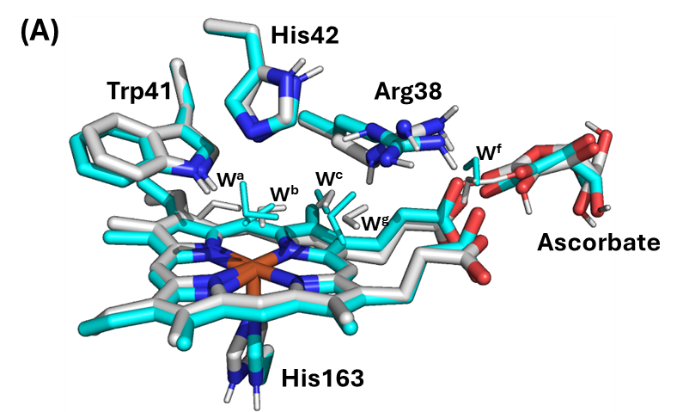 | 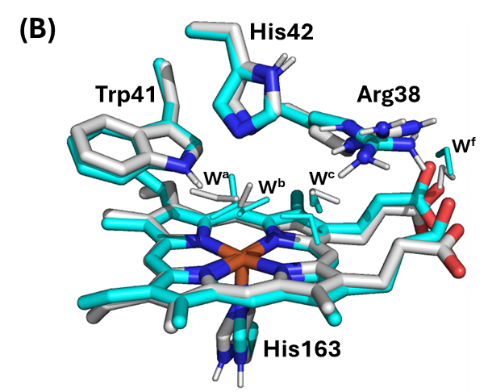 |
| --- | --- |
| **Figure S2**. Superposition of DFT (B3LYP-D3) optimized geometries (cyan) with (A) the neutron structure of the ferric APX-ascorbate complex (with Arg38 in the neutral form, PDB 6XV4) and (B) the neutron structure of ferric APX (with Arg38 in the protonated form, PDB 6TAE). Some residues (Leu35, His169 and Arg172) included in the DFT calculations have been omitted, for clarity. | |

Table S1 shows the relative energies of the 3 tautomers of Arg38, within the protein environment as described by the cluster model. In these calculations, the initial positions of the water molecules were taken from the neutron structure^[1]^, and their positions did not change significantly after geometry optimization (0.3 Å RMSD for both neutral and charged arginine systems, hydrogen atoms and water molecules not considered).

| **Table S1.** Relative energies (kcal/mol) of the different tautomers of Arg38 in the cluster model. | | | |
| --- | --- | --- | --- |
| **Tautomer**  **Multiplicity** | **1** | **2** | **3** |
| **2** | 0.00 | 20.90 | 0.50 |
| **4** | 23.13 | 28.94 | 23.70 |
| **6** | 1.97 | 29.43 | 2.68 |

1.2 Proton affinities. To evaluate proton affinities, we calculated the energy difference between the neutral and charged forms and balanced the system with a change of protonation of a solvent molecule, as shown in equations 1-3; the solvent molecule does not interact with the model-Arg system, and the energy of the water and hydronium molecules is calculated at the same level of theory as in the cluster model calculations.

Arg38-neutral + H^+^ ® Arg38-charged (1)

H_3_O^+^ ® H_2_O + H^+^ (2)

Arg38-neutral + H_3_O^+^ ® Arg38-charged + H_2_O (3)

First, as a validation of the level of theory used, the same calculations were performed for a guanidine molecule, because the experimental (gas phase) proton affinity for this molecule (and for water) are available.^[11]^ If protonation of guanidine is as represented by equation 1, the corresponding experimental proton affinity is 236.0 kcal/mol;^[11]^ adding the proton affinity of water with an inverted sign (–165.3 kcal/mol^[11]^, as equation 2 is the reverse process for protonation of water), together yielding an experimental value for equation 3 of 70.7 kcal/mol. DFT calculations of guanidine and water at the B3LYP-D3/6-31G(d,p) level of theory yield an energy of 74.2 kcal/mol which is reasonably close to the experimental value. Hence, we consider this level of theory adequate for the present purposes.

We considered an initial model of arginine as zwitterionic, but a proton moved from the NH_3_^+^ to the COO^–^ at the very early stage of the geometry optimization, leading to formation of the non- zwitterionic form of the molecule. Here we used the extended conformation of the arginine and followed the same procedure as for guanidine molecule. There are reported proton affinities and gas basicity for arginine, obtained theoretically^[12-15]^ and experimentally,^[11, 16]^ but all of them report values of Δ*H* around –250 kcal/mol. Here we have used the experimental value reported in NIST^[11]^ which, after adding the negative value for the proton affinity of water, yields a value of 86.1 very similar to the value calculated here, 81.9, see Table 1in the main text.

Table S2 shows the calculated proton affinity calculated in the gas phase (vacuum) and using a polarizable continuum model (PCM) of solvent of water and a value of dielectric constant ε = 4 for guanidine, an arginine amino acid, and Arg38 within the cluster model described in the main text.

| **Table S2.** Calculated proton affinity* of guanidinium, arginine and Arg38 within the protein (kcal/mol)* in vacuum, and in a polarizable continuum model (PCM). | | | |
| --- | --- | --- | --- |
|  | Guanidine | Arginine | Active site model** |
| experiment (gas phase) | 70.7 | 86.1 | -- |
| vacuum | 74.2 | 81.9 | 137.0 |
| PCM water | 49.5 | 50.7 | 51.9 |
| PCM ε=4 | 64.6 | 58.5 | 50.9 |
| * As defined by equation 3.  ** Cluster model as described in the main text; and using B3LYP-D3/6-31G(d,p) multiplicity = 2. | | | |

1.3 QM(DFT)/MM calculations. We applied DFT/MM calculations^[17]^ to further test the effects of the extended protein environment on proton affinity, and to quantify specific proton movements in this single proton pathway. Such DFT/MM calculations can provide accurate structures (including electronic structures) of heme enzymes, complementing experimental investigations and testing empirical MM models.^[18-20]^ Starting structures for the DFT/MM geometry optimizations were taken randomly from ten frames of a MM molecular dynamics trajectory, described in the next section. The QM region includes 196 (neutral arginine) or 197 (charged arginine) atoms (link atoms not included): the heme group, the side chains of residues Leu35, Arg38, Trp41, His42, His163, His169, Arg172 and Trp179, the ascorbate and five water molecules. Similar QM partitions have been previously tested to study heme systems, particularly peroxidases but the QM partition presented here is, to our knowledge, one of the largest to date. An electrostatic embedding scheme^[21]^ was used in all the calculations, and hydrogen link atoms were used to treat the QM/MM boundaries.

QM/MM calculations were performed at the DFT level of QM theory. After equilibration of the system by MM-MD as described above, it was trimmed to a sphere of 30 Å radius centred on the Fe atom. Atoms further than 20 Å from the iron were kept fixed during the QM/MM calculations. No cutoffs were used for the non-bonding MM and QM/MM interactions. QM/MM geometry optimizations were performed, with the QM sub-system described by the B3LYP hybrid functional and the 6-31G(d,p) basis set^[22]^  for all atoms except for iron (iron was described by the LANL2DZ basis set/effective core potential^[23]^). QM dispersion corrections were included using the DFT-D3 method as implemented in the Q-Chem v5.0 software package.^[24-25]^ This combination of functional and basis set has been used successfully in iron-containing enzymes.^[5-9]^ An electrostatic embedding scheme ^[21]^ was used in all the calculations and hydrogen link atoms were used to treat the QM/MM boundaries. As in the previous DFT calculations, all three possible multiplicity states were considered. Table S3 shows the relative energies of the different tautomers of Arg38 within the protein environment described by the QM/MM calculations.

| **Table S3.** Relative energies (kcal/mol) of the different tautomers of Arg38 in the QM/MM calculations. | | | |
| --- | --- | --- | --- |
| **Tautomer**  **Multiplicity** | **1** | **2** | **3** |
| **2** | 3.4 | 27.8 | 0.0 |
| **4** | 6.0 | 24.3 | 2.9 |
| **6** | 3.2 | 30.7 | 1.9 |

We also evaluated the proton affinities of Arg38 within the protein environment using the procedure as described above for the DFT cluster model. Table S4 presents the proton affinities for different randomly selected frames, as well as the average values for the various multiplicities. These calculations were performed using tautomer 1, as this is the form reported in the crystal structure and used in the molecular dynamics simulations.

| **Table S4.** Calculated proton affinities* of Arg38 within the full-length protein at QM/MM level (kcal/mol)*.Tautomer, as found crystallographically, was used for these calculations. | | | |
| --- | --- | --- | --- |
|  | Multiplicity | | |
|  | 2 | 4 | 6 |
| frame 1 | 181.78 | 205.65 | 208.56 |
| frame 2 | 209.73 | 158.85 | 203.08 |
| frame 3 | 205.17 | 158.80 | 186.40 |
| frame 4 | 240.03 | 182.62 | 192.66 |
| frame 5 | 239.67 | 220.02 | 209.76 |
| frame 6 | 159.80 | 49.63 | 114.14 |
| frame 7 | 99.24 | 173.01 | 161.96 |
| frame 8 | 219.59 | 234.16 | 163.08 |
| frame 9 | 218.49 | 159.96 | 199.21 |
| frame 10 | 217.88 | 208.89 | 234.62 |
| average | 199.14 | 175.16 | 187.35 |
| *As defined by equation 3 at B3LYP-D3/6-31G(d,p) level of theory. | | | |

These proton affinity values, as well as those obtained from DFT cluster model calculations, are dependent on the relative energies, which in turn are influenced by the positions and orientations of the water molecules (as previously mentioned for the tautomers). The intention here is to test whether the protein environment shifts the proton affinity to favour a neutral arginine. The results indicate that the system is more stable in the protonated form and should predominantly exist in that state.

1.4 Molecular dynamics (MD) simulations. The starting geometry was set up from the neutron crystal structure of ascorbate peroxidase in complex with ascorbate (PDB code 6XV4).^[1]^ All ionizable residues were in their standard protonation states according to the HBUILD routine in CHARMM.^[26]^ Histidine tautomers were determined based on local hydrogen bonding networks and they were all singly protonated on N^δ1^. The system was then solvated with a pre-equilibrated TIP3P cubic water box of 75 Å^3^ volume. To neutralise the system, a KCl concentration corresponding to 0.15 M was used; randomly picked water molecules distant from the protein were replaced by K^+^ and Cl^−^ ions. 10000 steps of energy minimization, followed by molecular dynamics simulations using periodic boundary conditions, were carried out. One production trajectory in the NPT ensemble was performed for 500 ns each, after 10 ns of equilibration, using NAMD.^[27]^ A time step of 2 fs was used. Temperature and pressure were held constant at 300K and 1 atm, respectively, using Langevin dynamics and the Nosé-Hoover Langevin piston pressure control with default NAMD settings. The internal structure of solvent molecules was held rigidly with the SHAKE constraint.^[28]^ We used the CHARMM36^[29]^ force field, and the particle mesh Ewald method^[30]^ for long-range electrostatics, with a 12 Å cutoff for the evaluation of (direct space) nonbonded interactions. Parameters for the heme group in its (ferric) oxidized state were obtained from Autenrieth *et al*,^[31]^ the parameters for the corresponding neutral tautomer of arginine were obtained from Li *et al*,^[32]^ and parameters for the ascorbate were obtained using the CHARMM General Force Field program (CGenFF).^[33]^ To check how local geometry around the active centre differ in structure we calculated time-averaged RMSD values for the QM region (very similar to the cluster model) across all frames for each system, considering only heavy atoms and no waters. All RMSD values were approximately 1 Å, indicating the QM region's geometry remains relatively stable throughout the MD simulations. We then: 1- Generated an "averaged" geometry for each MD simulation 2- Selected the frame closest to this average (since averaged geometries can be unphysical) 3- Compared all four systems by calculating inter-trajectory RMSDs.

The results (Table S5) show that the RMSD differences between systems are comparable to the time-averaged RMSD within each trajectory. This means the cluster geometries (excluding H atoms and waters) don't vary significantly across different tautomeric and protonation states.

| **Table S5.** Trajectory-Averaged and Inter-Trajectory RMSD of QM Region. | | | | |
| --- | --- | --- | --- | --- |
|  | Tautomers and protonation states | | | |
|  | T1 prot | T1 neut | T2 prot | T3 prot |
| RMSD_(time-avg)_^1^ | 1.07 | 1.04 | 0.89 | 1.04 |
| RMSD_(inter-traj)_^2^ | 0.74 | 0.73 | 0.77 | 0.77 |
| ^1^ Average RMSD over trajectory. For each MD run, average the RMSD of the QM region over all frames.  ^2^ Inter-trajectory RMSD of QM region. RMSD calculated between the representative QM structures of different MD runs, reflecting how much the QM region geometry differs across trajectories. | | | | |

1.5 Identification of proton delivery channels. MD Analysis. We performed water-chain identification analysis on MD simulations of ascorbate peroxidase with protonated and neutral Arg38 (PDB entries 6XV4 and 6TAE (data collected at 100 K)), as well as cytochrome c peroxidase (CcP, PDB: 4CVI (data collected at 100K)) and horseradish peroxidase (HRP, PDB: 1H5A (data collected at 100K)), all in the ferric state**.** After the MD simulations (1.5 μs per system), a reference point is defined 2 Å away from the Fe atom and aligned to form a 180° angle with the N atom of the proximal His, approximating the center of the active site. Next, we searched for any water molecule within a 3 Å sphere centered on this point. For each water molecule found, an additional 3 Å sphere is defined to locate a connected water molecule. This process is repeated for each subsequent water molecule until a chain of at least five water molecules is established. In this context, a "chain" is defined as a series of water molecules where each is less than 3 Å apart from its neighbor, effectively mapping potential water pathways from the active site toward any direction. This analysis was performed using the Visual Molecular Dynamics (VMD) software.^[34]^ To automate the process, custom Tcl scripts were developed and are provided as part of this SI.

This preliminary analysis was performed using a trajectory of only 300 frames, trimmed from a 1.5 μs simulation at regular intervals. While analyzing additional frames might reveal more water chains, this approach provides a reasonable indication of the most probable water pathways, see Figure 3 in the main text.

Analysis of hydrogen bonding in the simulations shows approximately conserved positions for some water molecules, especially within the active site. We define water chains based on spatial proximity, requiring that waters are within 3.5 Å of each other (between heavy atoms), applying standard MD analysis criteria for hydrogen bonding. Water molecules are rotating, and dynamically forming and breaking hydrogen bonds over time; this chain of appropriately spaced waters is sufficient to support proton transfer (as in bulk water in appropriate conditions).

CAVER analysis of the crystal structure using a default 0.9 Å probe radius revealed a potential wateronly pathway in the γ direction. However, MD simulations, show that such purely water-mediated chains from the reaction centre to the γ direction are rare. Instead, what is frequently observed is a composite pathway involving Arg38. That is, the pathway proceeds as: reaction centre → waters → Arg38 → waters → γ direction (or other directions e.g. distal).

We consider two scenarios based on our MD results:

Scenario 1: Comparison of purely water-only chains

Water-only chains extending from the reaction centre to the γ direction are rare. In contrast, water-only chains in the δ direction occur more frequently. Therefore, under this definition, the δ pathway appears more likely.

Scenario 2: Water chains involving Arg38 (γ direction) vs. water-only chains (δ direction)

Here, we quantify the number of MD frames that meet the following criteria:

• γ direction: One or more waters connecting reaction centre to Arg38, with at least three additional waters beyond Arg38.

• δ direction: A chain of at least five consecutive waters.

The number of frames meeting these criteria are:

Delta Gamma

AXP prot 100 111

AXP neut (all tautomers) 222 209

HRP 100 160

CcP 188 145

Frames were sampled at regular intervals for computational efficiency, and the definition of a water chain (e.g., the 3.5 Å cutoff) influences the results. Adjusting such parameters would change the absolute numbers, but the overall trend remains reasonably clear: the γ and δ directions are sampled with comparable frequency, depending on the system. Taken together, these results suggest that the Arg38-mediated pathway in the γ direction is a recurring feature across different enzymes, which may indicate functional relevance.

1.6 Identification of proton delivery channels. Channel (CAVER) Analysis. The CAVER software package^[35-36]^ identifies and characterizes tunnels and channels within protein structures by focusing primarily on the geometric properties of these pathways. It efficiently maps out routes from internal cavities to the surface, but it does not explicitly consider molecular interactions between the tunnel and passing molecules. To tailor the analysis for water, the probe radius parameter can be adjusted to approximate the size of a water molecule (typically around 1.4 Å). This setting directs CAVER to search for tunnels/channels that are sufficiently wide to accommodate water, highlighting potential pathways for water flow. On the other hand, a 0.9 Å probe radius is often used to detect narrow, functionally relevant pathways, such as those involved in proton transfer, where water must pass through tight spaces. While 1.4 Å reflects the typical size of a water molecule, 0.9Å captures confined environments, making it suitable for identifying selective transport routes or catalytic gorges in dynamic enzymes.^[37]^ CAVER analysis can be applied to both static structures (such as crystal structures) and MD simulation trajectories, capturing how channels open or close in response to protein conformational changes.

We performed channel identification analysis on MD simulations of ascorbate peroxidase featuring both protonated and neutral Arg38 (PDB entries 6XV4 and 6TAE), as well as cytochrome c peroxidase (C*c*P, PDB: 4CVI) and horseradish peroxidase (HRP, PDB: 1H5A), all in the ferric state. Using a 1.4 Å radius probe, we identified a well-defined channel connecting the heme iron (Fe) center to the protein surface (solvent) along the *δ*-direction in all systems. This suggests the channel is sufficiently wide to permit water flux, rather than being restricted to a single-file water chain. This is consistent with the overlay of the MD-obtained chain of consecutive water molecules (corresponding to Figure 3A in the main text) and the CAVER-identified δ-channel (corresponding to Figure 4 in the main text), as shown in Figure S3. As can be seen, the agreement is excellent

| 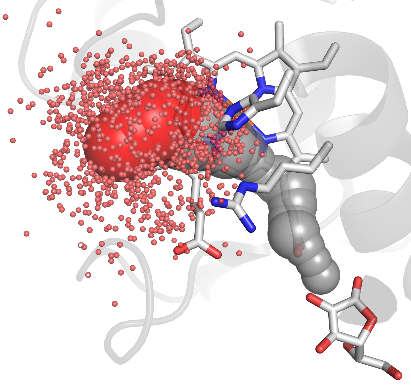 |
| --- |
| **Figure S3**. CAVER identified channels and superposed MD-obtained water chains in APX. |

Notably, a channel along the γ-direction was only detectable when using a smaller 0.9 Å probe. This implies that while water flow is less likely in this direction, the pathway is structurally compatible with proton hopping (e.g., via the Grotthuss mechanism) through a transient single-file water wire and/or polar residues (*e.g*., Arg38 in APX).

1.7 QM(DFTB2)/MM calculations. QM/MM MD simulations were carried out at the self-consistent charge density functional tight binding (DFTB2)^[38]^ (approximate density functional theory) quantum mechanical level. The transfer of a proton from a hydronium ion placed in the solvent along the δ-direction to the distal His42 through bridging water molecules was simulated using adiabatic mapping^[17]^ and subsequently umbrella sampling at the DFTB2/MM level as implemented in CHARMM^[39]^. The reaction paths were scanned by performing restrained geometry optimizations along the reaction coordinate *z* = *r*_1_(O_water_–H_water_) – *r*_2_(N_Hisε2_–H_water_), using a step size of 0.1 Å both in the forward and backward directions to obtain hysteresis-free results^[40]^, see Figure S4. . The QM region for all of these calculations included all 47 atoms depicted in black in Figure S4, *i.e.* including the side chains of His42, Trp41, 6 water molecules and a H_3_O^+^ ion.

| 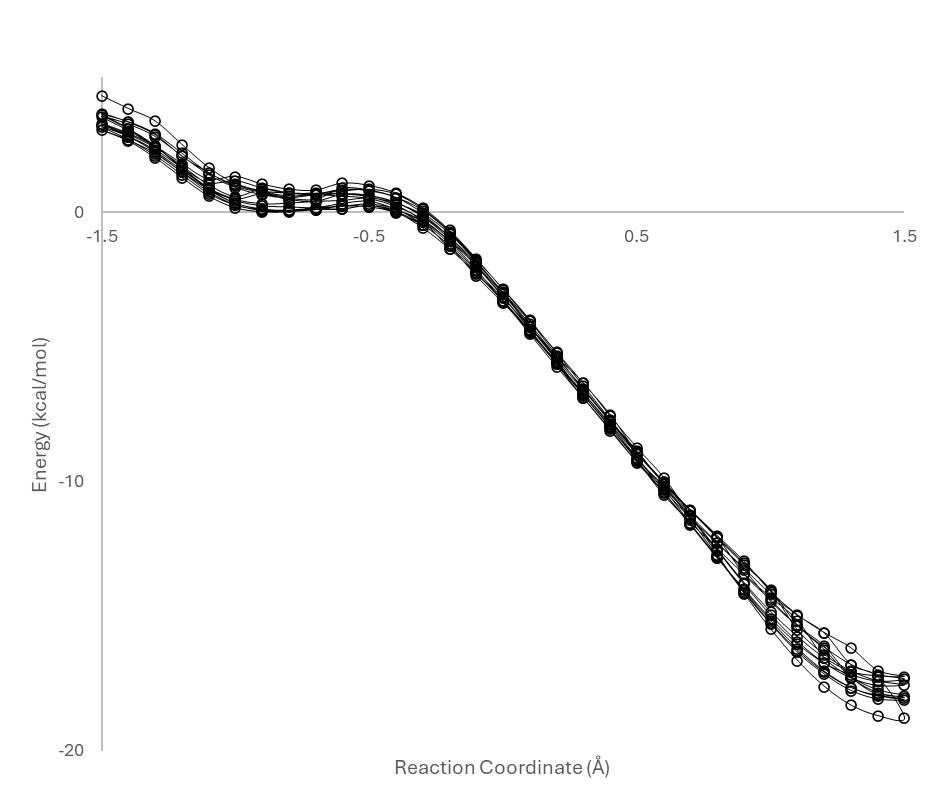 |
| --- |
| **Figure S4.** Forward and backwards potential energy scans at DFTB2/MM level of theory of the proton transfer from a hydronium ion in the solvent bulk to the distal histidine. Each point indicates a partial minimization at a reaction coordinate value. |

Umbrella sampling molecular dynamics free energy calculations were performed starting from each of the 25 minimized geometries of the last round of adiabatic mapping. The umbrella sampling simulations were used to compute the potential of mean force along the reaction coordinate described above. We have successfully and extensively used this protocol in previous studies of other enzymatic systems.^[41-44]^ We used the same level of theory (DFTB2), reaction coordinate and QM region as described above. 25 umbrella windows were run, placing harmonic biasing potentials along the reaction coordinate using a spring constant of 300 kcal/(mol Å^2^). The QM/MM biased molecular dynamics on each window was run for 10ps after equilibration using a Langevin thermostat and a time step of 1 fs. Two different algorithms were used to calculate the free energies. We used a high-precision implementation of the dynamic histogram analysis method (DHAM)^39-41^ to calculate the free energies and the kinetic rate of the reaction. We also evaluated the free energies using a binless implementation of the weighted histogram analysis method (WHAM).^[45-46]^

The QM/MM umbrella sampling simulations at the DFTB2^[38]^ quantum mechanical level were used to examine how readily a proton can access the reaction centre from the solvent along the δ-direction. These calculations test only whether a proton can access the active site directly from the solvent, and for this for this reason we drastically reduced the QM region compared to the previous DFT QM/MM calculations (main manuscript), excluding Arg38 and the heme, and retaining His42, Trp41, six water molecules, and an H_3_O^+^ ion towards δ-direction, as depicted in Figure S5

| 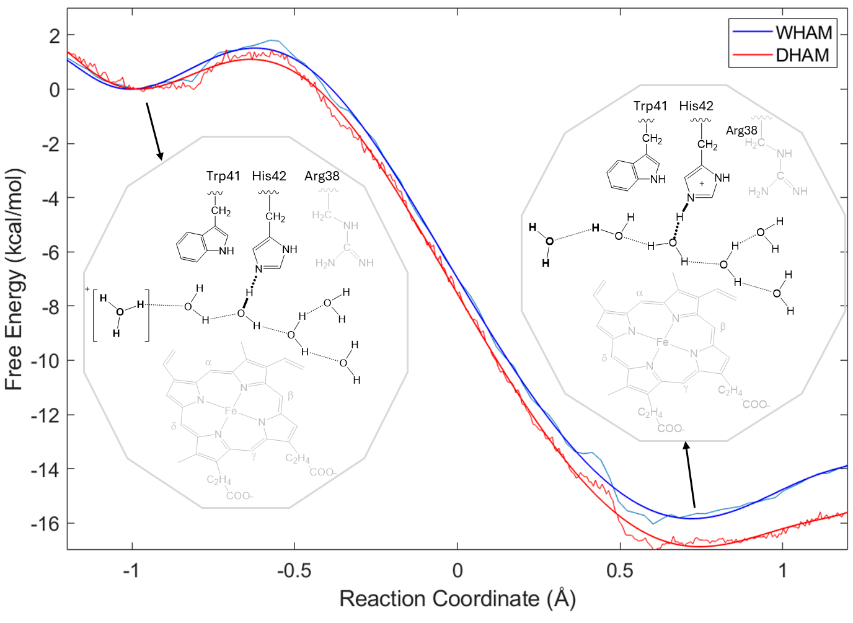 |
| --- |
| **Figure S5.** Alternative proton source mechanism. Free energy reaction profile of proton transfer from a hydronium ion (left) to the distal histidine (His42), obtained from umbrella sampling calculations. 2D representations of the reactant and product states are shown. Black atoms were treated by QM. Light grey atoms, shown for orientation, were treated by MM together with the rest of the protein and solvent. Two algorithms were used to calculate the free energies, WHAM (blue line) and DHAM (red line). |

The simulations show that proton transfer from the hydronium has a reaction barrier lower than 2 kcal/mol and a reaction energy of approximately –16 kcal/mol from these calculations. This very low barrier might reduce close to zero if we consider zero-point effects,^[47-48]^ suggesting that once the hydronium ion is in the active site, its lifetime would be very short in favour of a new state with a protonated histidine, as expected. These results shows that an alternative mechanism for delivery of a proton to the heme is feasible and does not depend on the ascorbate. Instead, proton delivery can occur through rapid exchange with the solvent at the δ-heme edge, followed by transfer from the solvent bulk to the active site, specifically to His42. Protein atomic positions do not change significantly on proton transfer and the positions of water molecules are well maintained and consistent with their positions in the crystal structures and previous DFT and DFT/MM calculations. These results confirm that, although individual water molecules exchange dynamically, specific positions in the crystal structure remain consistently occupied by water molecules, a pattern reproduced in the MD simulations. The conservation of these positions suggests a potential catalytic or other functional role for these water molecules within the protein.

1.8 Local electric field (LEF) calculations. Enzyme electric fields play a very important role in heme ezyme catalysis.^[49-50]^ To quantify the LEF in APX and other heme peroxidases, we employed the TUPÃ code^[51]^, a computational tool designed to calculate electric field magnitude and vectors at user-defined positions throughout an MD trajectory, based on the atomic partial charges derived from force fields and coordinates of the system. We performed 500 ns MM MD simulations for each APX system in the protonated and neutral forms of Arg38 and quantified electric fields for multiple snapshots (every 100 ps) from the MD simulations (Figures 5A and S6). The LEFs were computed at three key points; (a) at the location of the water molecule W^f^, (b) at the midpoint between W^f^ and the C^z^ atom of Arg38, and (c) at the C^z^ atom of Arg38, along the conserved γ-channel. The labeling scheme follows that for Figure 1. For all other heme peroxidases, atomic coordinates were obtained from their respective crystal structures in the Protein Data Bank (PDB), and the LEFs were subsequently calculated for each structure for generalisation across the enzyme family. Point charges were derived from the force field, and the TUPÃ code computed LEF vectors and magnitudes using Coulomb’s law, considering contributions from all atoms in the enzyme. Notably, solvent (*e.g*., water or ions) was not explicitly included in the electric field calculations, so the reported LEFs reflect the intrinsic field generated by the protein scaffold alone. The parameters for heme compound I/II were generated using MCPB.py in AMBER.

| 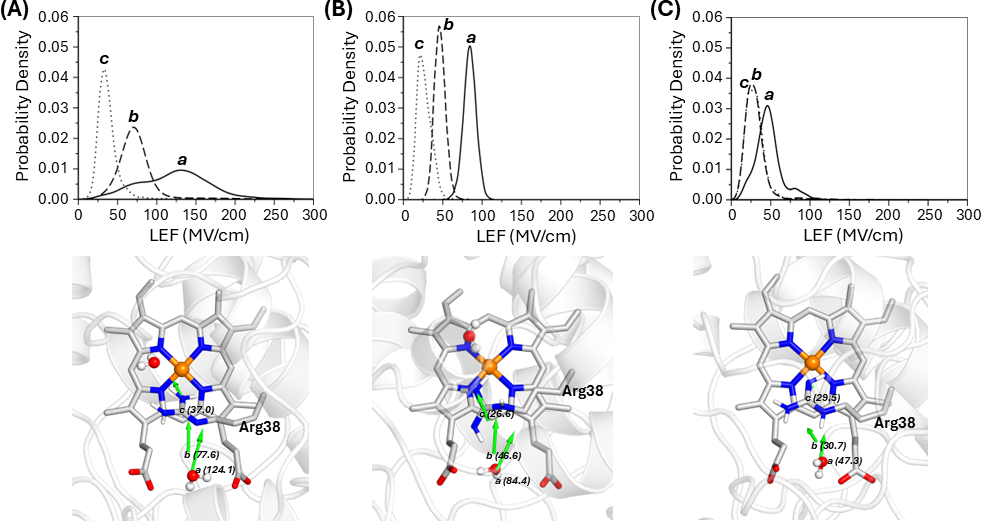 |
| --- |
| **Figure S6.** Local electric field (LEF) profiles along the γ-channel of APX for three neutral tautomers, Figure S1, of Arg38: (A) tautomer 1, (B) tautomer 2, and (C) tautomer 3. The top graphs show probability density distributions of LEF magnitudes (in MV/cm) calculated at three key positions along the γ-channel for each tautomer of neutral Arg38. The curves labelled a, b, and c in each graph correspond to the LEF values at (a) at the location of the water molecule W^f^, (b) at the midpoint between W^f^ and the C^z^ atom of Arg38, and (c) at the C^z^ atom of Arg38, respectively. The labelling scheme is the same as in Figure 1. Differences in the LEF magnitudes across the neutral tautomers, compared to the corresponding LEFs shown in Figure 5A for protonated Arg38, highlight the effect of the charge on Arg38 on the electrostatic environment near the heme iron. The bottom panels in each case show the LEF magnitudes (shown in black numerals) overlaid on the structures of the γ-channel. Green arrows indicate the direction, and the length of the arrow indicates the relative magnitude of the LEF vectors along the pathway, in all cases pointing from the γ-heme edge towards the heme iron. Structural variations among the tautomers alter the magnitude of LEF along the channel (compare the values at points a, b and c); however, the LEF vectors consistently point toward the heme iron, indicating a preserved electrostatic steering effect pointing in the direction of the heme iron from the γ-heme edge. |

1.9 Visualisation of electric field lines in virtual reality (VR). Visualizing the complex three-dimensional Local Electric Fields (LEFs) vectors within heme peroxidases poses significant challenges in a traditional 2D format, where electric field lines and their spatial orientations can be cluttered and difficult to interpret, especially in dense protein environments like channels. This limitation can obscure critical insights into how electric fields guide proton delivery to the heme active site. To address this, we used virtual reality (VR) for immersive and intuitive visualization.

| 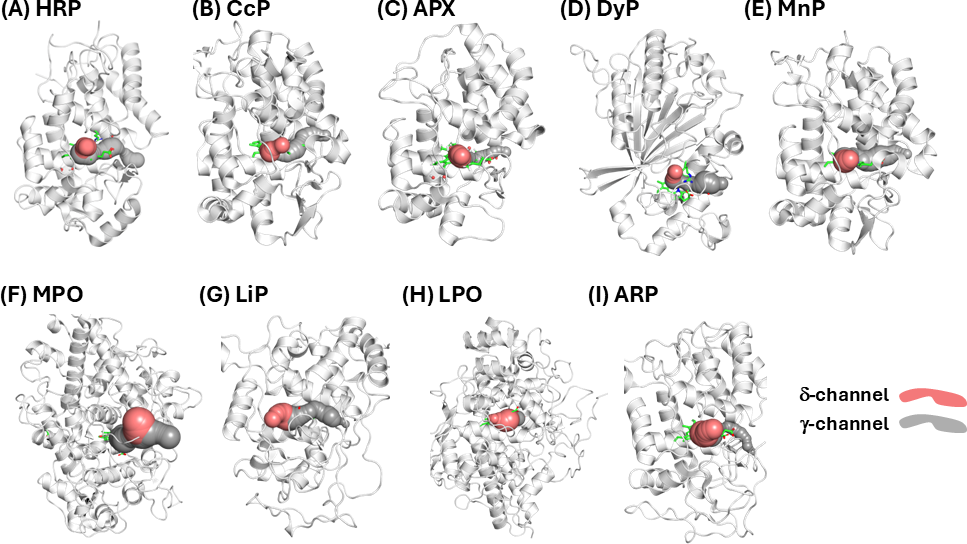 |
| --- |
| **Figure S7.** The solvent accessible γ− and δ-channel for different heme peroxidase crystal structures. (A) horseradish peroxidase (HRP, PDB code 1ATJ), (B) cytochrome c peroxidase, (CcP, 3R98), (C) ascorbate peroxidase (APX, 6XV4), (D) dye decolorizing peroxidase (DyP, 6KMN), (E) manganese peroxidase (MnP, 3M5Q), (F) myeloperoxidase (MPO, 1DNU), (G) lignin peroxidase (LiP, 1B85), (H) lactoperoxidase (LPO, 5B72), and (I) Arthromyces ramosus peroxidase (ARP, 1ARP). Proteins are depicted in a cartoon representation (white), with the δ-channel in orange and the γ-channel in gray, as identified from CAVER analysis. |

Both the γ- and δ-channels were identified in APX and other peroxidase structures studied here, confirming the presence of both channels across the enzyme family, Figure S7. We also examined the position of the conserved distal arginine across these structures, classifying them into “Arg-in” and “Arg-out” conformations depending on their spatial relationship with the heme-bound oxygen (see Table S6). The “in” and “out” conformations refer to the positional states of the conserved distal arginine (e.g., Arg38 in APX) relative to the heme group; where “in” indicates it is oriented toward the heme/ferryl oxygen, potentially forming hydrogen bonds, and “out” means a water molecule is present between the ferryl oxygen and the Arg.

| ***Table S6.*** *Classification of distal Arg conformations in Heme Peroxidase crystal structures* | |
| --- | --- |
| **Arg-in** | **Arg-out** |
| 1ATJ (HRP, ferric) | 6XV4 (APX, ferric) |
| 3M5Q (MnP, ferric) | 3R98 (CcP, ferric) |
| 6KMN (DyP, ferric) | 1B85 (LiP, ferric) |
| 1HCH (HRP, Compound I) | 1DNU (MPO, ferric) |
| 5EJT (CcP, Compound I) | 5B72 (LPO, ferric) |
| 1H55 (HRP, Compound II) | 1ARP (ARP, ferric) |
| 2XJ5 (CcP, Compound II) | 2XI6 (APX, Compound I) |
|  | 5JPR (APX, Compound II) |

Electrostatic potentials were then computed using the Adaptive Poisson-Boltzmann Solver (APBS)^[52]^ and mapped onto the protein surfaces to validate and rationalize the LEFs quantified by the TUPÃ, Table S7. In these calculations, implicit solvent effects were included by assigning a dielectric constant 78.0 and 4.0 for the water solvent and to the protein, respectively, and a physiological ionic strength, 0.15 M, using monovalent ions. A linearized Poisson–Boltzmann equation was used with a smoothed molecular surface, ensuring that both dielectric boundary effects and ionic screening were appropriately accounted for in the resulting electrostatic potential maps. Figure S8 shows the two-dimensional visualization of electric field lines along both channels for all of these proteins, rendered in PyMOL^[53]^. Electric field lines near the δ-channel, Figure S8A, can be visualised in a two-dimensional format because the δ-heme edge is exposed to solvent (so easily visible). This reveals an arrangement conducive to water transport and thereby, proton transfer, and the electric field lines leading towards the heme active site from the δ-heme edge are clearly observed. These lines appear directionally consistent, running from the positive to the negative region, suggesting a funnel-like electrostatic environment that could help stabilize and guide incoming positively charged proton or hydronium ions. Visualization of electric field lines along the γ-channel (Figure S8B) is significantly more complex because the trajectory of proton movement from the γ-edge to the heme active site through the interior of the protein is invisible when viewing the protein from the outside surface, so the two-dimensional view does not convey the spatial arrangement of electric field lines inside the protein molecule. The electric field lines in this case, Figure S8B, appear tangled and congested as they converge from inside the protein surface towards the heme iron, making it difficult to trace their paths or appreciate their three-dimensional trajectories.

| 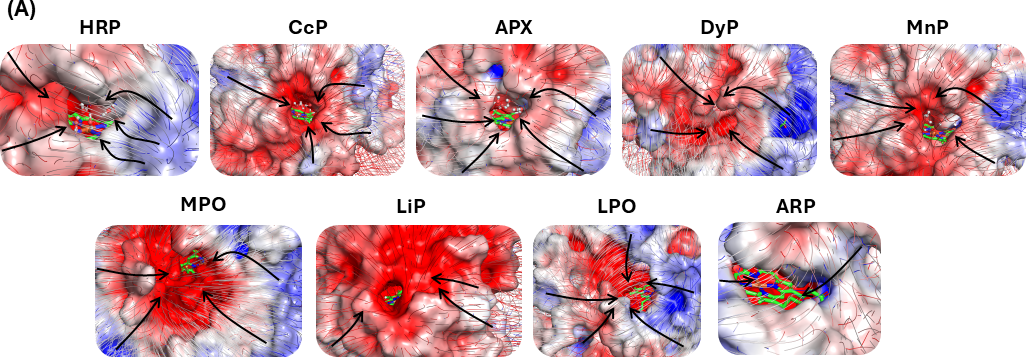  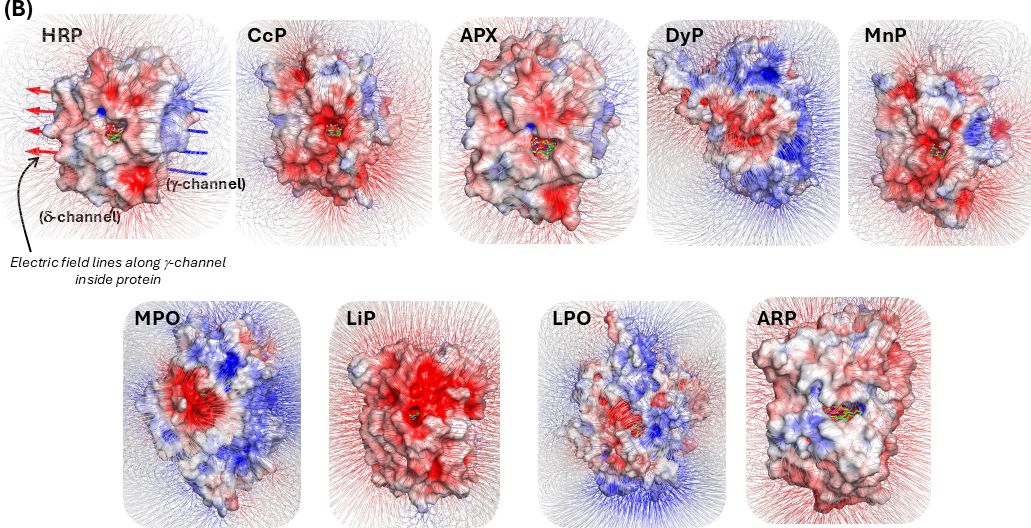 |
| --- |
| **Figure S8.** Electrostatic potentials and LEF lines in the δ- and γ-channels of APX and other heme peroxidases: horseradish peroxidase (HRP), cytochrome c peroxidase (CcP), dye-decolorizing peroxidase (DyP), manganese peroxidase (MnP), myeloperoxidase (MPO), lignin peroxidase (LiP), lactoperoxidase (LPO), and A. ramosus peroxidase (ARP). (A) Electric field lines along the δ-channel pathway, with the black arrows depicting a schematic representation of electric field lines near the δ-channel, illustrating LEF directionality toward the heme active site. (B) Visualization of electric field lines along the γ-channel pathway (depicted from right to left through the interior of the protein). The arrows represent the electric field lines travelling inside the protein along the γ-channel. The direction of electric field lines is from positive (blue) to negative (red) electrostatic potential. The protein surfaces are coloured accordingly by electrostatic potential. The electric field lines reveal how electrostatic environments shape the electric field towards the heme iron via the δ- and γ-channels, facilitating and driving proton transfer along these pathways. |

To address this, and to improve the visualization of the fields, we imported the LEF data calculated from APBS (including field lines and electrostatic potential) into UnityMol^[54]^, a molecular visualization platform optimized for VR. In this VR environment, field lines were rendered as 3D streamlines, allowing us to interactively navigate the protein structure and observe the directional properties of the electric fields from multiple perspectives (see movies S3-S5). This immersive approach provided a clearer, more intuitive understanding of the spatial distribution of electric fields across different peroxidase structures, revealing how electric fields align with the δ- and γ-channel to facilitate proton transfer. Crucially, these structural and electrostatic insights were not fully discernible from 2D projections alone, underscoring the added value of VR for interpreting complex electrostatic landscapes in biomolecules.

| ***Table S7.*** *Quantitative analysis of LEF strengths (in MV/cm) at different points along the γ-channel across heme peroxidase crystal structures.* | | | | | | |
| --- | --- | --- | --- | --- | --- | --- |
| Heme Peroxidase | LEF (in MV/cm) at each point | | | | | |
|  | W^f **^ | W^f^-W | W | W^f^-R^***^ | W-R | R^****^ |
| HRP | 138.08 | 97.05 | 91.86 | 52.69 | 39.49 | 30.06 |
| HRP-I | 176.73 | 134.22 | 79.33 | 60.18 | 54.70 | 34.35 |
| HRP-II | 181.58 | 138.88 | 85.13 | 61.93 | 55.91 | 35.67 |
| CcP | 94.12 | NaN | NaN | 70.29 | NaN | 52.61 |
| CcP-I | 81.03 | 73.61 | 63.09 | 57.22 | 55.11 | 32.23 |
| CcP-II | 85.47 | 80.35 | 65.24 | 59.72 | 55.30 | 32.00 |
| APX | 88.31 | NaN | NaN | 75.56 | NaN | 39.64 |
| APX-I | 96.36 | NaN | NaN | 80.13 | NaN | 49.52 |
| APX-II | 202.23 | NaN | NaN | 89.24 | NaN | 44.63 |
| DyP | 206.11 | 94.53 | 94.61 | 76.71 | 73.32 | 65.10 |
| LmP | 80.90 | NaN | NaN | 75.99 | NaN | 73.03 |
| MPO | 112.62 | NaN | NaN | 98.56 | NaN | 91.47 |
| LPO | 111.92 | 109.69 | 100.54 | 99.44 | 77.02 | 71.10 |
| ARP | 111.07 | NaN | NaN | 75.66 | NaN | 49.55 |
| MnP | 127.57 | NaN | NaN | 77.73 | NaN | 60.09 |
| *NaN indicates that no water at this point was present in the crystal structure.  ** Point a in Figure 5A.  *** Point b in Figure 5A.  **** Point c in Figure 5A. | | | | | | |

Table S7 summarizes the LEF magnitudes calculated at five defined positions along the γ-channel for APX and all the other heme peroxidases studied, including resting state, Compound I, and Compound II structures. The points of LEF calculation were selected based on conserved locations of structured water molecules and the arginine residue along the γ-channel, as observed in APX and structurally aligned across other peroxidases. The positions include: (i) W^f^ (surface water molecule), (ii) W^f^–W midpoint, (iii) W (intermediate water), (iv) W^f^–R midpoint, (v) W–R midpoint, and (vi) R (conserved distal arginine, e.g. Arg38 in APX), Figure 5B. The data clearly shows a general trend across enzymes: in all cases, the LEF magnitude decreases progressively from the solvent-accessible surface toward the heme iron, supporting the presence of an electrostatic funnel that may guide proton transfer. Notably, the table also reveals differences in absolute field strengths and gradients between enzymes. These variations suggest that while the directionality of the field is conserved, the degree of electrostatic preorganisation may differ across these enzymes, potentially reflecting functional tuning of proton delivery mechanisms.

# 1.10 Coordinates

Cartesian coordinates of the optimised geometries are provided for both the cluster model and the QM/MM full system. In addition, Gaussian input and output files corresponding to the geometry optimisations of the cluster model for the different tautomers are included. For the QM/MM calculations, CHARMM-format Cartesian coordinates, topology and parameter files, PSF files, as well as all necessary input files required for execution and reproducibility are provided for all studied tautomers.

# 1.11 References

[1] H. Kwon, J. Basran, J. M. Devos, R. Suardíaz, M. W. Van Der Kamp, A. J. Mulholland, T. E. Schrader, A. Ostermann, M. P. Blakeley, P. C. E. Moody, E. L. Raven,"*Visualizing the protons in a metalloenzyme electron proton transfer pathway"* *Proc. Natl. Acad. Sci. U.S.A.* **2020**, *117*, 6484-6490.

[2] A. D. Becke,"*Density-functional thermochemistry. III. The role of exact exchange"* *Journal of Chemical Physics* **1993**, *98*, 5648-5652.

[3] M. J. Frisch, G. W. Trucks, H. B. Schlegel, G. E. Scuseria, M. A. Robb, J. R. Cheeseman, G. Scalmani, V. Barone, B. Mennucci, G. A. Petersson, H. Nakatsuji, M. Caricato, X. Li, H. P. Hratchian, A. F. Izmaylov, J. Bloino, G. Zheng, J. L. Sonnenberg, M. Hada, M. Ehara, K. Toyota, R. Fukuda, J. Hasegawa, M. Ishida, T. Nakajima, Y. Honda, O. Kitao, H. Nakai, T. Vreven, J. A. Montgomery, J. E. Peralta, F. Ogliaro, M. Bearpark, J. J. Heyd, E. Brothers, K. N. Kudin, V. N. Staroverov, R. Kobayashi, J. Normand, K. Raghavachari, A. Rendell, J. C. Burant, S. S. Iyengar, J. Tomasi, M. Cossi, N. Rega, J. M. Millam, M. Klene, J. E. Knox, J. B. Cross, V. Bakken, C. Adamo, J. Jaramillo, R. Gomperts, R. E. Stratmann, O. Yazyev, A. J. Austin, R. Cammi, C. Pomelli, J. W. Ochterski, R. L. Martin, K. Morokuma, V. G. Zakrzewski, G. A. Voth, P. Salvador, J. J. Dannenberg, S. Dapprich, A. D. Daniels, Farkas, J. B. Foresman, J. V. Ortiz, J. Cioslowski, D. J. Fox, Wallingford CT, **2009**.

[4] S. Grimme, S. Ehrlich, L. Goerigk,"*Effect of the damping function in dispersion corrected density functional theory"* *Journal of computational chemistry* **2011**, *32*, 1456-1465.

[5] S. Kalita, S. Shaik, H. K. Kisan, K. D. Dubey,"*A Paradigm Shift in the Catalytic Cycle of P450: The Preparatory Choreography during O2 Binding and Origins of the Necessity for Two Protonation Pathways"* *ACS Catal.* **2020**, *10*, 11481-11492.

[6] R. Suardíaz, L. Masgrau, J. M. Lluch, À. González-Lafont,"*On the Regio- and Stereospecificity of Arachidonic Acid Peroxidation Catalyzed by Mammalian 15-Lypoxygenases: A Combined Molecular Dynamics and QM/MM Study"* *Chemphyschem* **2013**, *14*, 3777-3787.

[7] R. Suardíaz, L. Masgrau, J. M. Lluch, À. González-Lafont,"*Regio- and Stereospecificity in the Oxygenation of Arachidonic Acid Catalyzed by Leu597 Mutants of Rabbit 15-Lipoxygenase: A QM/MM Study"* *Chemphyschem* **2014**, *15*, 2303-2310.

[8] R. Suardíaz, L. Masgrau, J. M. Lluch, À. González-Lafont,"*Introducing Mutations to Modify the C13/C9 Ratio in Linoleic Acid Oxygenations Catalyzed by Rabbit 15-Lipoxygenase: A QM/MM and MD Study"* *Chemphyschem* **2014**, *15*, 4049-4054.

[9] J. M. Grandner, R. A. Cacho, Y. Tang, K. N. Houk,"*Mechanism of the P450-Catalyzed Oxidative Cyclization in the Biosynthesis of Griseofulvin"* *ACS Catal.* **2016**, *6*, 4506-4511.

[10] J. Rak, P. Skurski, J. Simons, M. Gutowski,"*Low-energy tautomers and conformers of neutral and protonated arginine"* *J. Am. Chem. Soc.* **2001**, *123*, 11695-11707.

[11] E. P. L. Hunter, S. G. Lias,"*Evaluated Gas Phase Basicities and Proton Affinities of Molecules: An Update"* *Journal of Physical and Chemical Reference Data* **1998**, *27*, 413-656.

[12] C. Bleiholder, S. Suhai, B. Paizs,"*Revising the proton affinity scale of the naturally occurring α-amino acids"* *Journal of the American Society for Mass Spectrometry* **2006**, *17*, 1275-1281.

[13] M. Rožman,"*Proton affinity of several basic non-standard amino acids"* *Chemical Physics Letters* **2012**, *543*, 50-54.

[14] A. Moser, K. Range, D. M. York,"*Accurate proton affinity and gas-phase basicity values for molecules important in biocatalysis"* *J Phys Chem B* **2010**, *114*, 13911-13921.

[15] S. Gronert, D. C. Simpson, K. M. Conner,"*A reevaluation of computed proton affinities for the common α-amino acids"* *Journal of the American Society for Mass Spectrometry* **2009**, *20*, 2116-2123.

[16] G. Bouchoux, S. Desaphy, S. Bourcier, C. Malosse, R. N. B. Bimbong,"*Gas-Phase Protonation Thermochemistry of Arginine"* *The Journal of Physical Chemistry B* **2008**, *112*, 3410-3419.

[17] R. Lonsdale, J. N. Harvey, A. J. Mulholland,"*A practical guide to modelling enzyme-catalysed reactions"* *Chem. Soc. Rev.* **2012**, *41*, 3025-3038.

[18] S. Chatterjee, J. Nochebuena, G. A. Cisneros,"*Impact of an Ionic Liquid Solution on Horseradish Peroxidase Activity"* *J. Am. Chem. Soc.* **2024**, *146*, 13247-13257.

[19] M. Ansari, S. Bhattacharjee, D. A. Pantazis,"*Correlating Structure with Spectroscopy in Ascorbate Peroxidase Compound II"* *J. Am. Chem. Soc.* **2024**, *146*, 9640-9656.

[20] J. N. Harvey, C. M. Bathelt, A. J. Mulholland,"*QM/MM modeling of compound I active species in cytochrome P450, cytochrome C peroxidase, and ascorbate peroxidase"* *J. Comput. Chem.* **2006**, *27*, 1352-1362.

[21] H. L. Woodcock Iii, M. Hodošček, A. T. B. Gilbert, P. M. W. Gill, H. F. Schaefer Iii, B. R. Brooks,"*Interfacing Q-Chem and CHARMM to perform QM/MM reaction path calculations"* *J. Comput. Chem.* **2007**, *28*, 1485-1502.

[22] P. C. Hariharan, J. A. Pople,"*The influence of polarization functions on molecular orbital hydrogenation energies"* *Theoretica chimica acta* **1973**, *28*, 213-222.

[23] P. J. Hay, W. R. Wadt,"*Ab initio effective core potentials for molecular calculations. Potentials for the transition metal atoms Sc to Hg"* *The Journal of Chemical Physics* **1985**, *82*, 270-283.

[24] Y. Shao, Z. Gan, E. Epifanovsky, A. T. B. Gilbert, M. Wormit, J. Kussmann, A. W. Lange, A. Behn, J. Deng, X. Feng, D. Ghosh, M. Goldey, P. R. Horn, L. D. Jacobson, I. Kaliman, R. Z. Khaliullin, T. Kuś, A. Landau, J. Liu, E. I. Proynov, Y. M. Rhee, R. M. Richard, M. A. Rohrdanz, R. P. Steele, E. J. Sundstrom, H. L. Woodcock, P. M. Zimmerman, D. Zuev, B. Albrecht, E. Alguire, B. Austin, G. J. O. Beran, Y. A. Bernard, E. Berquist, K. Brandhorst, K. B. Bravaya, S. T. Brown, D. Casanova, C.-M. Chang, Y. Chen, S. H. Chien, K. D. Closser, D. L. Crittenden, M. Diedenhofen, R. A. DiStasio, H. Do, A. D. Dutoi, R. G. Edgar, S. Fatehi, L. Fusti-Molnar, A. Ghysels, A. Golubeva-Zadorozhnaya, J. Gomes, M. W. D. Hanson-Heine, P. H. P. Harbach, A. W. Hauser, E. G. Hohenstein, Z. C. Holden, T.-C. Jagau, H. Ji, B. Kaduk, K. Khistyaev, J. Kim, J. Kim, R. A. King, P. Klunzinger, D. Kosenkov, T. Kowalczyk, C. M. Krauter, K. U. Lao, A. D. Laurent, K. V. Lawler, S. V. Levchenko, C. Y. Lin, F. Liu, E. Livshits, R. C. Lochan, A. Luenser, P. Manohar, S. F. Manzer, S.-P. Mao, N. Mardirossian, A. V. Marenich, S. A. Maurer, N. J. Mayhall, E. Neuscamman, C. M. Oana, R. Olivares-Amaya, D. P. O’Neill, J. A. Parkhill, T. M. Perrine, R. Peverati, A. Prociuk, D. R. Rehn, E. Rosta, N. J. Russ, S. M. Sharada, S. Sharma, D. W. Small, A. Sodt, T. Stein, D. Stück, Y.-C. Su, A. J. W. Thom, T. Tsuchimochi, V. Vanovschi, L. Vogt, O. Vydrov, T. Wang, M. A. Watson, J. Wenzel, A. White, C. F. Williams, J. Yang, S. Yeganeh, S. R. Yost, Z.-Q. You, I. Y. Zhang, X. Zhang, Y. Zhao, B. R. Brooks, G. K. L. Chan, D. M. Chipman, C. J. Cramer, W. A. Goddard, M. S. Gordon, W. J. Hehre, A. Klamt, H. F. Schaefer, M. W. Schmidt, C. D. Sherrill, D. G. Truhlar, A. Warshel, X. Xu, A. Aspuru-Guzik, R. Baer, A. T. Bell, N. A. Besley, J.-D. Chai, A. Dreuw, B. D. Dunietz, T. R. Furlani, S. R. Gwaltney, C.-P. Hsu, Y. Jung, J. Kong, D. S. Lambrecht, W. Liang, C. Ochsenfeld, V. A. Rassolov, L. V. Slipchenko, J. E. Subotnik, T. Van Voorhis, J. M. Herbert, A. I. Krylov, P. M. W. Gill, M. Head-Gordon,"*Advances in molecular quantum chemistry contained in the Q-Chem 4 program package"* *Molecular Physics* **2015**, *113*, 184-215.

[25] Y. Shao, L. F. Molnar, Y. Jung, J. Kussmann, C. Ochsenfeld, S. T. Brown, A. T. B. Gilbert, L. V. Slipchenko, S. V. Levchenko, D. P. O’Neill, R. A. DiStasio Jr, R. C. Lochan, T. Wang, G. J. O. Beran, N. A. Besley, J. M. Herbert, C. Yeh Lin, T. Van Voorhis, S. Hung Chien, A. Sodt, R. P. Steele, V. A. Rassolov, P. E. Maslen, P. P. Korambath, R. D. Adamson, B. Austin, J. Baker, E. F. C. Byrd, H. Dachsel, R. J. Doerksen, A. Dreuw, B. D. Dunietz, A. D. Dutoi, T. R. Furlani, S. R. Gwaltney, A. Heyden, S. Hirata, C.-P. Hsu, G. Kedziora, R. Z. Khalliulin, P. Klunzinger, A. M. Lee, M. S. Lee, W. Liang, I. Lotan, N. Nair, B. Peters, E. I. Proynov, P. A. Pieniazek, Y. Min Rhee, J. Ritchie, E. Rosta, C. David Sherrill, A. C. Simmonett, J. E. Subotnik, H. Lee Woodcock Iii, W. Zhang, A. T. Bell, A. K. Chakraborty, D. M. Chipman, F. J. Keil, A. Warshel, W. J. Hehre, H. F. Schaefer Iii, J. Kong, A. I. Krylov, P. M. W. Gill, M. Head-Gordon,"*Advances in methods and algorithms in a modern quantum chemistry program package"* *Physical Chemistry Chemical Physics* **2006**, *8*, 3172-3191.

[26] J. Huang, A. D. MacKerell Jr,"*CHARMM36 all-atom additive protein force field: Validation based on comparison to NMR data"* *J. Comput. Chem.* **2013**, *34*, 2135-2145.

[27] J. C. Phillips, D. J. Hardy, J. D. C. Maia, J. E. Stone, J. V. Ribeiro, R. C. Bernardi, R. Buch, G. Fiorin, J. Hénin, W. Jiang, R. McGreevy, M. C. R. Melo, B. K. Radak, R. D. Skeel, A. Singharoy, Y. Wang, B. Roux, A. Aksimentiev, Z. Luthey-Schulten, L. V. Kalé, K. Schulten, C. Chipot, E. Tajkhorshid,"*Scalable molecular dynamics on CPU and GPU architectures with NAMD"* *The Journal of Chemical Physics* **2020**, *153*, 044130.

[28] H. C. Andersen,"*Rattle: A “velocity” version of the shake algorithm for molecular dynamics calculations"* *Journal of Computational Physics* **1983**, *52*, 24-34.

[29] J. Huang, S. Rauscher, G. Nawrocki, T. Ran, M. Feig, B. L. de Groot, H. Grubmüller, A. D. MacKerell,"*CHARMM36m: an improved force field for folded and intrinsically disordered proteins"* *Nat. Methods* **2017**, *14*, 71-73.

[30] T. Darden, D. York, L. Pedersen,"*Particle mesh Ewald: An N⋅log(N) method for Ewald sums in large systems"* *The Journal of Chemical Physics* **1993**, *98*, 10089-10092.

[31] F. Autenrieth, E. Tajkhorshid, J. Baudry, Z. Luthey-Schulten,"*Classical force field parameters for the heme prosthetic group of cytochrome c"* *J. Comput. Chem.* **2004**, *25*, 1613-1622.

[32] L. Li, I. Vorobyov, A. D. MacKerell, T. W. Allen,"*Is Arginine Charged in a Membrane?"* *Biophysical Journal* **2008**, *94*, L11-L13.

[33] K. Vanommeslaeghe, E. Hatcher, C. Acharya, S. Kundu, S. Zhong, J. Shim, E. Darian, O. Guvench, P. Lopes, I. Vorobyov, A. D. Mackerell Jr.,"*CHARMM general force field: A force field for drug-like molecules compatible with the CHARMM all-atom additive biological force fields"* *J. Comput. Chem.* **2010**, *31*, 671-690.

[34] W. Humphrey, A. Dalke, K. Schulten,"*VMD: Visual molecular dynamics"* *Journal of Molecular Graphics* **1996**, *14*, 33-38.

[35] E. Chovancova, A. Pavelka, P. Benes, O. Strnad, J. Brezovsky, B. Kozlikova, A. Gora, V. Sustr, M. Klvana, P. Medek, L. Biedermannova, J. Sochor, J. Damborsky,"*CAVER 3.0: A Tool for the Analysis of Transport Pathways in Dynamic Protein Structures"* *PLoS Comput. Biol.* **2012**, *8*, e1002708.

[36] A. Pavelka, E. Sebestova, B. Kozlikova, J. Brezovsky, J. Sochor, J. Damborsky,"*CAVER: Algorithms for Analyzing Dynamics of Tunnels in Macromolecules"* *IEEE/ACM Trans. Comput. Biol. Bioinform.* **2016**, *13*, 505-517.

[37] D. Sehnal, R. Svobodová Vařeková, K. Berka, L. Pravda, V. Navrátilová, P. Banáš, C.-M. Ionescu, M. Otyepka, J. Koča,"*MOLE 2.0: advanced approach for analysis of biomacromolecular channels"* *J. Cheminform.* **2013**, *5*, 39.

[38] M. Elstner,"*The SCC-DFTB method and its application to biological systems"* *Theor. Chem. Acc.* **2006**, *116*, 316-325.

[39] B. R. Brooks, C. L. Brooks, 3rd, A. D. Mackerell, Jr., L. Nilsson, R. J. Petrella, B. Roux, Y. Won, G. Archontis, C. Bartels, S. Boresch, A. Caflisch, L. Caves, Q. Cui, A. R. Dinner, M. Feig, S. Fischer, J. Gao, M. Hodoscek, W. Im, K. Kuczera, T. Lazaridis, J. Ma, V. Ovchinnikov, E. Paci, R. W. Pastor, C. B. Post, J. Z. Pu, M. Schaefer, B. Tidor, R. M. Venable, H. L. Woodcock, X. Wu, W. Yang, D. M. York, M. Karplus,"*CHARMM: the biomolecular simulation program"* *J. Comput. Chem.* **2009**, *30*, 1545-1614.

[40] E. Rosta, H. L. Woodcock, B. R. Brooks, G. Hummer,"*Artificial reaction coordinate “tunneling” in free-energy calculations: The catalytic reaction of RNase H"* *J. Comput. Chem.* **2009**, *30*, 1634-1641.

[41] S. L. Dürr, O. Bohuszewicz, D. Berta, R. Suardiaz, P. G. Jambrina, C. Peter, Y. Shao, E. Rosta,"*The Role of Conserved Residues in the DEDDh Motif: the Proton-Transfer Mechanism of HIV-1 RNase H"* *ACS Catal.* **2021**, *11*, 7915-7927.

[42] M. A. L. Limb, R. Suardíaz, I. M. Grant, A. J. Mulholland,"*Quantum Mechanics/Molecular Mechanics Simulations Show Saccharide Distortion is Required for Reaction in Hen Egg-White Lysozyme"* *Chem. Eur. J.* **2019**, *25*, 764-768.

[43] P. Saura, R. Suardíaz, L. Masgrau, À. González-Lafont, E. Rosta, J. M. Lluch,"*Understanding the Molecular Mechanism of the Ala-versus-Gly Concept Controlling the Product Specificity in Reactions Catalyzed by Lipoxygenases: A Combined Molecular Dynamics and QM/MM Study of Coral 8R-Lipoxygenase"* *ACS Catal.* **2017**, *7*, 4854-4866.

[44] R. Suardíaz, P. G. Jambrina, L. Masgrau, À. González-Lafont, E. Rosta, J. M. Lluch,"*Understanding the Mechanism of the Hydrogen Abstraction from Arachidonic Acid Catalyzed by the Human Enzyme 15-Lipoxygenase-2. A Quantum Mechanics/Molecular Mechanics Free Energy Simulation"* *J. Chem. Theory Comput.* **2016**, *12*, 2079-2090.

[45] S. Kumar, J. M. Rosenberg, D. Bouzida, R. H. Swendsen, P. A. Kollman,"*THE weighted histogram analysis method for free-energy calculations on biomolecules. I. The method"* *J. Comput. Chem.* **1992**, *13*, 1011-1021.

[46] E. Rosta, M. Nowotny, W. Yang, G. Hummer,"*Catalytic Mechanism of RNA Backbone Cleavage by Ribonuclease H from Quantum Mechanics/Molecular Mechanics Simulations"* *J. Am. Chem. Soc.* **2011**, *133*, 8934-8941.

[47] A. J. Mulholland, W. G. Richards,"*Modeling Enzyme Reaction Intermediates and Transition States:  Citrate Synthase"* *The Journal of Physical Chemistry B* **1998**, *102*, 6635-6646.

[48] M. W. van der Kamp, F. Perruccio, A. J. Mulholland,"*Ab initio QM/MM modelling of acetyl-CoA deprotonation in the enzyme citrate synthase"* *Journal of Molecular Graphics and Modelling* **2007**, *26*, 676-690.

[49] D. Bím, A. N. Alexandrova,"*Local Electric Fields As a Natural Switch of Heme-Iron Protein Reactivity"* *ACS Catal.* **2021**, *11*, 6534-6546.

[50] S. A. Siddiqui, T. Stuyver, S. Shaik, K. D. Dubey,"*Designed Local Electric Fields─Promising Tools for Enzyme Engineering"* *JACS Au* **2023**, *3*, 3259-3269.

[51] M. D. Poleto, J. A. Lemkul,"*TUPA: Electric field analyses for molecular simulations"* *J. Comput. Chem.* **2022**, *43*, 1113-1119.

[52] E. Jurrus, D. Engel, K. Star, K. Monson, J. Brandi, L. E. Felberg, D. H. Brookes, L. Wilson, J. Chen, K. Liles, M. Chun, P. Li, D. W. Gohara, T. Dolinsky, R. Konecny, D. R. Koes, J. E. Nielsen, T. Head-Gordon, W. Geng, R. Krasny, G. W. Wei, M. J. Holst, J. A. McCammon, N. A. Baker,"*Improvements to the APBS biomolecular solvation software suite"* *Protein Sci.* **2018**, *27*, 112-128.

[53] W. L. DeLano,"*The PyMOL Molecular Graphics System"* *DeLano Scientific, San Carlos, CA, USA* **2002**.

[54] X. Martinez, M. Baaden,"*UnityMol prototype for FAIR sharing of molecular-visualization experiences: from pictures in the cloud to collaborative virtual reality exploration in immersive 3D environments"* *Acta Crystallogr D Struct Biol* **2021**, *77*, 746-754.
